# The effect of macrophage polarization on the expression of the oxytocin signalling system in enteric neurons

**DOI:** 10.1186/s12974-021-02313-w

**Published:** 2021-11-08

**Authors:** Yao Shi, Shuang Li, Haojie Zhang, Jianchun Zhu, Tongtong Che, Bing Yan, Jingxin Li, Chuanyong Liu

**Affiliations:** 1 Department of Physiology, School of Basic Medical Sciences, Cheeloo Medical College, Shandong University, 44 Wenhua Xi Road, Jinan, 250012 Shandong People’s Republic of China; 2 School of Biological Science and Technology, Jinan University, 336 Nanxinzhuang Xi Road, Jinan, 250012 People’s Republic of China; 3 Jinan Central Hospital Affiliated to Shandong University, Jinan, 250012 Shandong People’s Republic of China; 4 Provincial Key Lab of Mental Disorders, Shandong University, Jinan, 250012 Shandong People’s Republic of China

**Keywords:** OT signalling system, Macrophage polarization, Proinflammatory and anti-inflammatory factors, Neural-immune interactions

## Abstract

**Background:**

The aim of the current study was to investigate the effect of macrophage polarization on the expression of oxytocin (OT) and the oxytocin receptor (OTR) in enteric neurons.

**Methods:**

In this study, we used a classic colitis model and D-mannose model to observe the correlation between macrophage polarization and OT signalling system. In order to further demonstrate the effect of macrophages, we examined the expression of OT signalling system after depletion of macrophages.

**Results:**

The data showed that, in vitro, following polarization of macrophages to the M1 type by LPS, the macrophage supernatant contained proinflammatory cytokines (IL-1β, IL-6 and TNF-α) that inhibited the expression of OT and OTR in cultured enteric neurons; following macrophage polarization to the M2 type by IL4, the macrophage supernatant contained anti-inflammatory cytokines (TGF-β) that promoted the expression of OT and OTR in cultured enteric neurons. Furthermore, M1 macrophages decreased the expression of the OT signalling system mainly through STAT3/NF-κB pathways in cultured enteric neurons; M2 macrophages increased the expression of the OT signalling system mainly through activation of Smad2/3 and inhibition of the expression of *Peg3* in cultured enteric neurons. In a colitis model, we demonstrated that macrophages were polarized to the M1 type during the inflammatory phase, with significant decreased in the expression of OT and OTR. When macrophages were polarized to the M2 type during the recovery phase, OT and OTR expression increased significantly. In addition, we found that D-mannose increased the expression of OT and OTR through polarization of macrophages to the M2 type.

**Conclusions:**

This is the first study to demonstrate that macrophage polarization differentially regulates the expression of OT and OTR in enteric neurons.

**Supplementary Information:**

The online version contains supplementary material available at 10.1186/s12974-021-02313-w.

## Introduction

Oxytocin (OT) is traditionally considered a nonapeptide hormone synthesized in the hypothalamus that is released from the posterior pituitary into circulation [1, 2] and is involved in milk let-down and uterine contraction [3]. Recent studies have shown that the oxytocin receptor (OTR) is widely present in the central and peripheral systems [3–5]. The role of OT is very broad; it not only participates in the regulation of certain social behaviours [6] but also relieves colitis through preventing the free radical damage cascade [7]. OT also promotes the proliferation and differentiation of adipose tissue-derived mesenchymal stem cells into nerve cells [8]. Studies in our laboratory and others have made clear that OT is an endogenous neuropeptide in the gastrointestinal tract and regulates the secretion, motility, and immunity of the digestive tract [9–11]. OT is mainly expressed in the myenteric nerve plexus and submucosal nerve plexus, including neuron bodies and nerve fibres [12].

The intestine is the largest immune organ in the body and contains various innate immune and adaptive immune cells. Macrophages are the most important innate immune cells [13] and are mainly distributed in the lamina propria and muscular layer. Lamina propria macrophages (LMs) are involved in the regulation of intestinal inflammation and have been identified as the main innate immune cells that induce inflammatory bowel disease (IBD) [14, 15]. Muscularis macrophages (MMs) are densely distributed and adjacent to enteric neurons and nerve fibres. There is a direct synaptic connection between macrophages and neurons [16]. This connection facilitates the dialogue between the enteric nervous system (ENS) and macrophages through neurotransmitters, growth factors, cytokines and hormones [17–19]. Tracey et al. proposed that the excitatory neurotransmitter acetylcholine targets resident macrophages to inhibit the production of inflammatory mediators [20]. Tissue-resident MMs protect enteric-associated neurons through the β2-adrenergic receptor and arginase 1 polyamine axis after infection [21].

We previously demonstrated that OT regulates the polarization of macrophages to relieve intestinal inflammation [22]. OTR-deficient mice are more susceptible to 2,4,6-trinitrobenzenesulfonic acid (TNBS)- and dextran sulfate sodium (DSS)-associated colitis [12]. Local and systemic levels of intestinal OT change during intestinal inflammation [9, 10]. Therefore, we assumed that inflammatory factors released by macrophages might regulate the expression of OT and OTR. This article aimed to explore whether the polarization of macrophages regulates the expression of the OT signalling system in the ENS, which might be of great significance for maintaining intestinal homeostasis.

## Materials and methods

### Reagents

Lipopolysaccharide was purchased from Sigma-Aldrich (St Louis, Ca). Recombinant mouse IL-4, recombinant mouse IL-6, recombinant mouse IL-1β, recombinant mouse TNF-α, recombinant mouse IL-10, and recombinant mouse TGF-β were purchased from R&D Systems (Minneapolis, MN). Tocilizumab, LY2109761, R-7050, Stattic, Bay 11-7082, and SIS3HCl were purchased from Selleck (Huston, USA). IL-1 receptor antagonist (IL1Ra) was purchased from Make Research Easy (Nanjing, China). Monoclonal mouse anti-β-actin (TA-09) was purchased from Zhongshan Golden Bridge Biotechnology (Beijing, China). Antibodies against OT, OTR, β-tubulin, CD206, F4-80, phospho-STAT3, STAT3, phospho-SMAD2, SMAD2, phospho-SMAD3, SMAD3, p65, and phospho-p65 were purchased from Abcam (Cambridge, UK) and Cell Signaling Technology (Danvers, MA). Secondary antibodies were purchased from Invitrogen Life Technology (Foster City, CA). Mouse ELISA kits were obtained from R&D Systems and CUSABIO (Wuhan, China). All reagents were analytical grade.

### Cell isolation and culture

The RAW264.7 macrophage-like cell line was purchased from the Cell Bank of the Chinese Academy of Science (Shanghai, China). Cells were cultured in Dulbecco’s modified Eagle’s medium (HyClone, Logan, Utah) supplemented with 10% heat-inactivated foetal bovine serum (Gibco, Foster City, CA) and 1% penicillin–streptomycin solution (Gibco) in a humidified incubator with 5% CO_2_ at 37 °C.

Enteric neurons were obtained from C57BL6/J mice as previously described [23]. Briefly, the mice were euthanized with no pain, and the outer skin of the peritoneum was cut. 3–5 cm of colon tissue was excised and immediately placed in a Sylgard™-lined dissecting dish containing carbogen-gassed Krebs saline. The composition of the Krebs saline was as follows (in mM): NaCl 120.6, KCl 5.9, CaCl_2_ 2.5, KH_2_PO_4_ 1.2, MgCl_2_ 1.2, and NaHCO_3_ 15.4. The mesentery was cut with Venus scissors, and the colonic segment was cut longitudinally along the mesentery. The colonic segment was fixed in a dissecting dish, and the contents were thoroughly washed. The mucosa, submucosa, serosal layer, and circular muscle layers were carefully removed with Venus forceps to expose the longitudinal muscle myenteric plexus (LMMP). The LMMP was incubated in Krebs saline containing papain (10 mg/ml) (P8150, Solarbio, Beijing, China) at 37 °C for 50 min. The LMMP was stretched as much as possible under the microscope. The LMMP was washed three times with 4 ml of ice-cold PBS and Dulbecco's modified Eagle's medium. Immediately thereafter, the LMMP was placed in Dulbecco’s modified Eagle’s medium (Gibco, Foster City, CA) containing collagenase II (1 mg/ml, Gibco), cut into pieces with scissors, and digested in a humidified incubator (Thermo Forma, Hamilton, USA) with 5% CO_2_ at 37 °C for 55 min. After digestion, the medium was added to terminate and centrifuged at 1000 RPM for 6 min. To culture enteric neurons, the sediment was collected and suspended in Dulbecco’s modified Eagle’s medium (Gibco, Foster City, CA) supplemented with 10% heat-inactivated foetal bovine serum (Gibco, Foster City, CA) and 1% penicillin–streptomycin solution (Gibco). The medium was replaced with fresh medium daily, and the cell growth status was carefully observed. On day 2 after digestion, we added cytarabine (Gibco, 5 μM) to the culture to inhibit the growth of glial cells and miscellaneous cells, and then pure cultured primary neurons were obtained.

### Experimental animals

Wild-type C57BL6/J mice were purchased from Beijing Vital River Laboratory Animal Technology and housed in a specific pathogen-free environment in the Animal Center of Shandong University. All mice in the experiment were aged 7–10 weeks and were randomly grouped. All experiments were performed at the Animal Center of Shandong and approved by the Medical Ethics Committee for Experimental Animals, Shandong University School of Basic Medicine Sciences (ECSBMSSDU2020-2-006). All measures were taken to minimize animal suffering.

### Immunocytofluorescence

The 24-well plate cell slides (coated with polylysine) or the LMMP were washed twice with cold PBS and were regularized with 4% paraformaldehyde at room temperature for 20 min, then washed three times with PBS for 15 min. The cell slides or the LMMP were handled with 3% hydrogen peroxide for 10 min and washed three times with PBS for 15 min. The cell slides or the LMMP were handled with 0.3% Triton-X100 for 60 min and washed three times with PBS for 15 min. After sealed with 10% donkey serum for 60 min at room temperature, immediately the cell slides or the LMMP were incubated with mouse anti-tubulin (1:800, Abcam), rabbit anti-OT (1:200, Invitrogen) primary antibodies diluted in blocking solution overnight at 4℃.

Paraffin sections of mouse colons were dewaxed for 2 h, soaked in xylene for 5 min, soaked in 100% alcohol, 95% alcohol, 90% alcohol, 75% alcohol, and 50% alcohol for 3 min each, and then rinsed with water for 3 min. Then, antigen retrieval was performed by boiling the slices in sodium citrate buffer (10 mM, pH 6, Beyotime) for 25 min. After cooling, the tissues were blocked with 10% donkey serum for 50 min at 25 °C and then incubated with mouse anti-Iba1(1:200, Abcam), mouse anti-tubulin (1:800, Abcam), rabbit anti-Arg1(1:200, Abcam), rabbit anti-F4-80 (1:200, Abcam) and mouse anti-iNOS (1:200, Abcam) primary antibodies diluted with blocking solution overnight at 4 °C. After three washes, the slides were incubated with Alexa Fluor 568 or 488 donkey anti-rabbit (1:1000, Invitrogen) or Alexa Fluor 488 donkey anti-mouse (1:1000, Invitrogen) secondary antibodies for 60 min, followed by counterstaining with DAPI (1:1000, Beyotime) for 5 min. Images were captured by a Laser Confocal Microscope (LSM980).

### RNA extraction and quantitative real-time PCR

Total RNA was extracted from neurons and LMMP by a tissue/cell rapid extraction kit (Spark jade, Qingdao, China). In short, lysis solution RL was added to lyse the cells and tissue fluid after homogenization to depolymerize and release protein and nucleic acid substances. Next, chloroform, absolute ethanol, protein-removing solution RE and rinsing solution RW were added in sequence to obtain approximately 20 μl of RNA solution. RNA was reverse-transcribed in a Takara PCR Thermal Cycler SP (Takara Bio, Shiga, Japan). Real-time quantitative PCR was carried out with SYBR Premix Dimer Eraser (Takara Bio, Shiga, Japan). Expression levels were normalized to the internal controls (β-actin), and the relative expression level was evaluated using the 2^−△△CT^ method. The primers were designed and verified by the Shanghai Genomics Institute (Shanghai, China), and the sequences of the primers are shown in Table 1.

**Table 1:** The primer sequences used in the study as follows

Name	Primer sequence (5' → 3')
β-actin	F primer: CTATTGGCAACGAGCGGTTCC
	R primer: CAGCACTGTGTTGGCATAGAG
TNFα	F primer: CCCTCACACTCAGATCATCTTCT
	R primer: GCTACGACGTGGGCTACAG
IL6	F primer: TCCTTCCTACCCCAATTCCA
	R primer: GTCTTGGTCCTTAGCCACTCC
IL1β	F primer: GGCAACCGTACCTGAACCCA
	R primer: CCACGATGACCGACACCACC
iNOS	F primer: CCGAAGCAAACATCACATTCA
	R primer: GGTCTAAAGGCTCCGGGCT
CCL2	F primer: TTAAAAACCTGGATCGGAACCAA
	R primer: GCATTAGCTTCAGTTACGGGT
Arg1	F primer: TGTCCCTAATGACAGCTCCTT
	R primer: GCATCCACCCAAATGACACAT
ChIL3	F primer: GATGGCCTCAACCTGGACTG
	R primer: CGTCAATGATTCCTGCTCCT
CD206	F primer: TGATTACGAGCAGTGGAAGC
	R primer: GCTACGACGTGGGCTACAG
OTR	F primer: GGCCGTGTTCCAGGTTCTC
	R primer: TGCAAGTATGACCAGACGAC
OT	F primer: ATCACCTACAGCGGATCTCAGAC
	R primer: CAGAGCCAGTAAGCCAAGCA
IL10	F primer: TAACTGCACCCACTTCCCAG
	R primer: TTGGCAACCCAAGTAACCCTTA
TGFβ	F primer: AGCTGCGCTTGCAGAGATTA
	R primer: AGCCCTGTATTCCGTCTCCT
Ym1	F primer: AGAAGGGAGTTTCAAACCTGGT
	R primer: GTCTTGCTCATGTGTGTAAGTGA

### ELISA

To assess levels of IL-1β, TNF-α, IL-6, TGF-β, and OT, enteric neuron and tissue supernatants were collected and centrifuged, and the concentrations of a specific protein were measured using a precoated ELISA kit according to the instruction manual.

### Colon explant culture

Colons from naive or colitis mice were flushed 10 times with PBS to remove faeces. Two 5-mm-long segments of colon tissue were cultured in 250 μl of RPMI supplemented with 10% heat-inactivated foetal bovine serum, 1% penicillin–streptomycin and 50 mg/ml gentamycin (Wako) in a 48-well plate (Corning, NY) at 37 °C for 30 min [24]. OT in the culture supernatant was measured by an ELISA kit.

### Western blotting analysis

Enteric neurons and tissues were harvested in ice-cold RIPA buffer (Bioster Bio, Pleasanton, CA) containing 1% PMSF and 1% phosphatase inhibitor (Bioster Bio, Pleasanton, CA). After stimulation with specific drugs, the cells were centrifuged at 13,800 × *g*/10 min at 4 °C. The supernatant was retained, and a BCA protein assay kit (Boster, USA) was used to quantify the total protein concentration. Next, the samples were mixed with 5 × loading buffer and boiled for 10 min at 100℃. Then, the proteins were loaded onto 15%, 10% and 8% gradient polyacrylamide gels and electrophoretically transferred to polyvinylidene difluoride membranes. The membranes were blocked with 5% nonfat dry milk for 90 min at 25 °C and then incubated with the following primary antibodies overnight at 4 °C: anti-OT (1:400, Invitrogen) antibody, anti-OTR (1:800, Abcam) antibody, anti-PEG3 (1:800, Abcam) antibody, anti-p65 (1:1000, CST) antibody, anti-phospho-p65 (1:800, CST) antibody, anti-STAT3 (1:1000, CST) antibody, anti-phospho-STAT3 (1:1000, CST) antibody, phospho-SMAD2 (1:400, CST) antibody, anti-SMAD2 (1:1000, CST) antibody, phospho-SMAD3 (1:500, CST) antibody, anti-SMAD3 (1:1000, CST) antibody and anti-β-actin (1:2000, Zhongshan Golden Bridge Biotechnology) antibody. After multiple washes, the membranes were incubated with secondary antibodies (1:2000, Beyotime) at room temperature for 50 min. The ECL kit reagent (MILLIPORE, USA) was used to generate chemiluminescence signals, which were captured with the Tanon Imaging System (Tanon-4600). The signal strength was analysed by ImageJ software.

### DSS colitis model

Acute colitis was induced by 2.5% DSS salt (reagent-grade, MW 36–50 kDa, MP Biomedicals, Canada) in drinking water for 7 days. In the second week, standard drinking water was available to the mice. The mice were randomly allocated into six groups: (1) control, (2) 1 W-DSS, (3) 1 W-DSS + 1 W-WATER, (4) 1 W-DSS + 2 W-WATER, (5) 1 W-DSS + 3 W-WATER, (6) 1 W-DSS + 6 W-WATER. Mice were euthanized by intraperitoneal administration of sodium pentobarbital (200 mg/kg) on days 7, 14, 21, 28 and 49. The distal colon was embedded in 4% paraformaldehyde solution, and the transverse sections were stained with haematoxylin and eosin. Histological scores were determined independently by two analysts who were blinded to the animal groupings. The assessment criteria included epithelial surface damage, crypt loss and inflammatory infiltrate as described by Zaki MH. The final score ranged from 0 to 6 (combining the inflammatory cell infiltration score and tissue damage score).

### D-Mannose model

Mice were fed 20% D-mannose (Sigma-Aldrich, St. Louis, MO) for 7 days [25, 26]. Clodronate liposomes (FormuMax, Silicon Valley, CA) were injected intraperitoneally one day before initiating the model and the third day after initiating the model to deplete macrophages; control liposomes were used as the control group [27–29]. The mice were randomly allocated into six groups: (1) control, (2) control + D-mannose, (3) control liposomes, (4) control liposomes + D-mannose, (5) clodronate liposomes, (6) clodronate liposomes + D-mannose. Distal colons were embedded in 4% paraformaldehyde solution, and transverse sections were stained with haematoxylin and eosin.

### Statistical analysis

Data are expressed as the mean ± SEM from at least two independent experiments. The number of samples in a specific experiment is reported. One-way ANOVA or two-tailed Student’s *t*-test was used for comparisons between groups. GraphPad Prism version 5 (La Jolla, CA) was used for statistical analysis. *P* < 0.05 was considered statistically significant.

## Results

### Effect of macrophage polarization on the OT signalling system

**Fig. 1:**
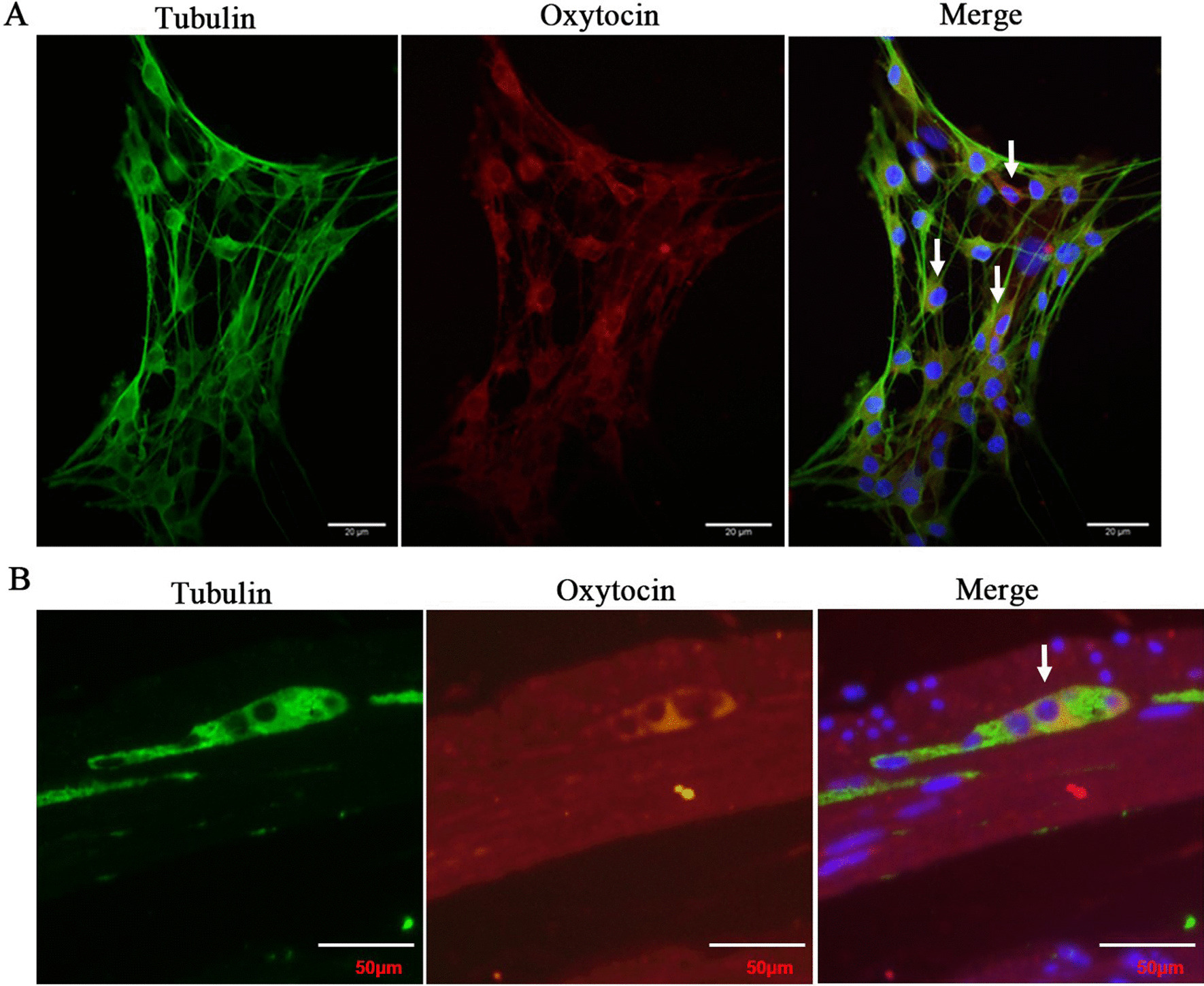
OT is mainly expressed in neuron bodies and nerve fibres. **A** Representative photographs of double immunofluorescent staining of tubulin (green) and oxytocin (OT) (red) within randomly captured images were obtained in cultured enteric neurons. Scale bar = 20 μm. **B** Representative photographs of double immunofluorescent staining of Tubulin (green) and Oxytocin (OT) (red) within randomly captured images were obtained in colon cross sections. Scale bar = 50 μm

**Fig. 2:**
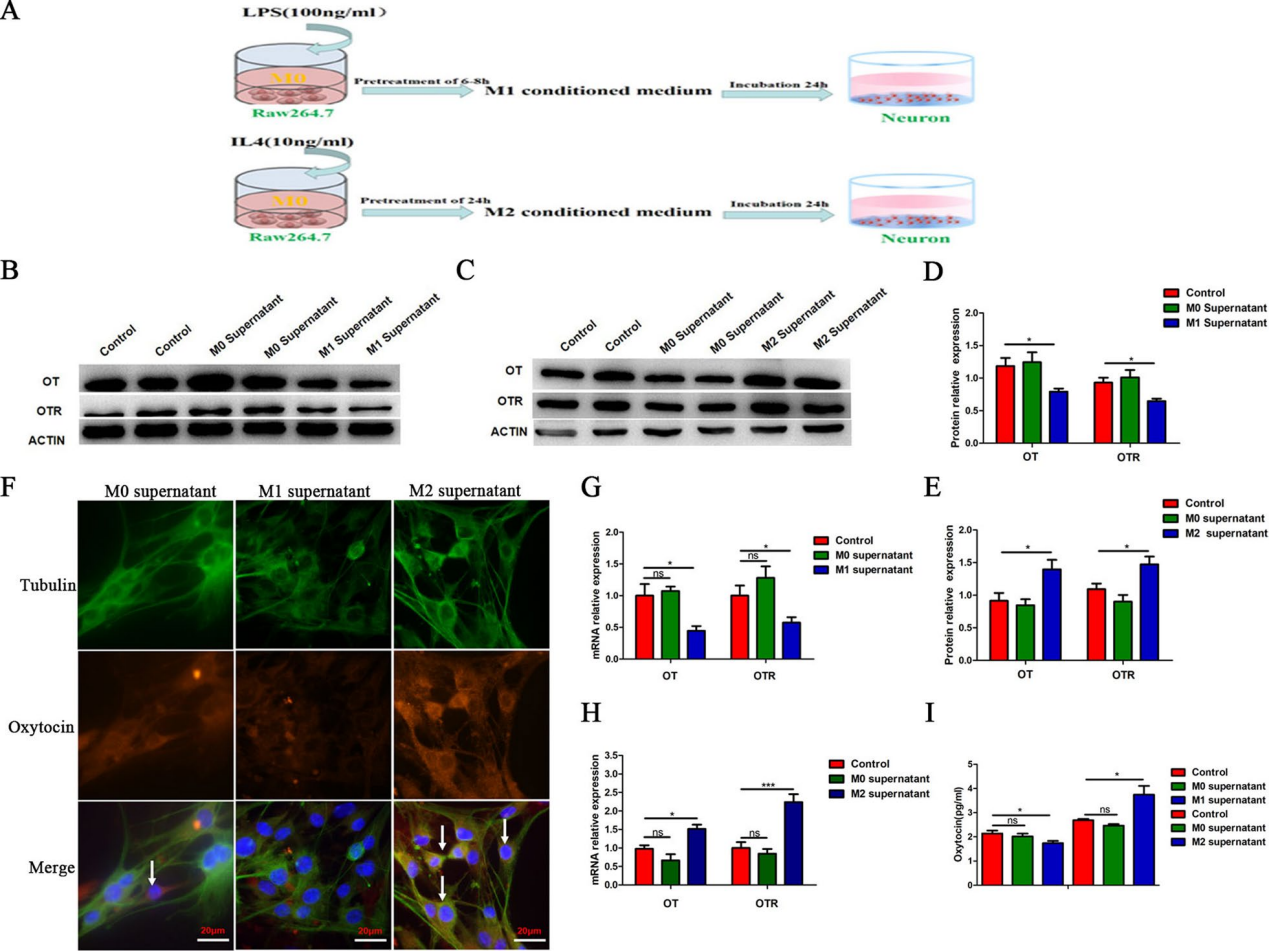
Effect of macrophage polarization on the OT signalling system. **A** Schematic diagram illustrating the procedures used to obtain conditioned medium. **B**–**E** The levels of OT and OTR proteins in cultured enteric neurons treated with or without conditioned medium were detected by western blot. M1 supernatant inhibited the protein expression of OT and OTR; by contrast, M2 supernatant promoted the protein expression of OT and OTR. **F** Representative photographs of double immunofluorescent staining of tubulin (green) and oxytocin (OT) (red) within randomly captured images were obtained in cultured enteric neurons. Scale bar = 20 μm. **G**, **H** The mRNA levels of OT and OTR in cultured enteric neurons treated with or without conditioned medium for 24 h were detected by qRT-PCR. M1 supernatant inhibited the mRNA levels of OT and OTR; by contrast, M2 supernatant promoted the mRNA levels of OT and OTR. **I** The expression of OT in enteric neurons treated with or without conditioned medium were detected by ELISA. The values represent the mean ± SEM of 6 samples and were compared by *t*-test or one-way ANOVA with Dunnett’s test for multiple comparisons. *p < 0.05, ***p < 0.001 vs. control group

To investigate the effect of macrophage polarization on the OT signalling system, we determined the position of OT in enteric neurons and further confirmed that OT was expressed in the bodies and nerve fibres (Fig. 1A, B). We used LPS or IL-4 to stimulate enteric neurons and observed no obvious changes in OT or OTR mRNA expression or OT secretion compared with the control group (Additional file 1: Fig. S1A–C). We used LPS to induce RAW264.7 cells to differentiate into M1 macrophages and collected the supernatant (M1 supernatant). We also induced RAW264.7 cells to differentiate into the M2 type with IL4 and collected the supernatant (M2 supernatant). We separately incubated enteric neurons with M1 supernatant, M2 supernatant or unstimulated macrophages supernatant (M0 supernatant) (Fig. 2A). In cells treated with M1 supernatant, the mRNA levels of OT and OTR were markedly decreased compared with those in the control group (Fig. 2G), whereas treatment with the M2 supernatant increased the mRNA expression of OT and OTR (Fig. 2H). This result was also verified in enteric neurons extracted from female mice (Additional file 1: Fig. S1E). Results from staining indicated that M1 supernatant inhibited the expression of OT, and M2 supernatant promoted the expression of OT compared with M0 supernatant in enteric neurons (Fig. 2F). The effects of the macrophage supernatant on OT and OTR protein expression were similar to those on mRNA expression (Fig. 2B, C, D, E, I).

### Effect of cytokines in macrophages supernatant on the expression of OT and OTR in enteric neurons

**Fig. 3:**
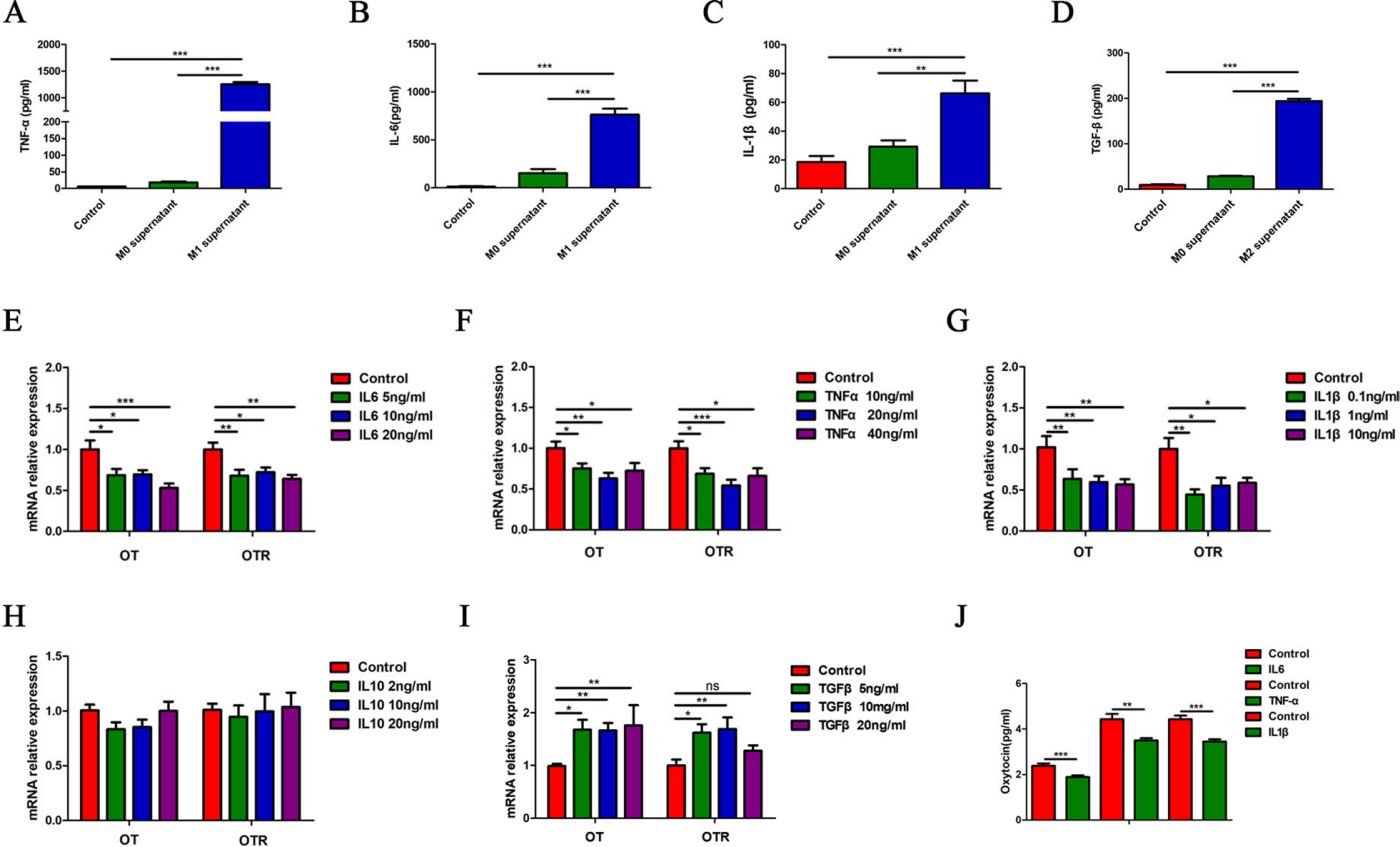
Effect of cytokines in macrophages supernatant on the expression of OT and OTR in enteric neurons. **A**–**C** M1 supernatant treatment for 24 h increased IL-6, TNF-α and IL-1β levels in cultured enteric neurons as assessed by ELISA. **D** M2 supernatant treatment for 24 h increased TGF-β levels in cultured enteric neurons as assessed by ELISA. **E**–**G** Stimulation of cultured enteric neurons for 6 h with IL-6 (0, 5, 10, 20 ng/ml), TNF-α (0, 10, 20, 40 ng/ml) or IL-1β (0, 0.1, 1, 10 ng/ml) decreased the mRNA expression of OT and OTR. **H** Stimulation of cultured enteric neurons with IL10 (0, 2, 10, 20 ng/ml) did not affect the expression of OT and OTR mRNA. **I** Stimulation of cultured enteric neurons with TGF-β (0, 5, 10, 20 ng/ml) for 6 h increased the mRNA expression of OT and OTR. **J** The concentration of OT was measured in cultured enteric neurons treated with or without IL-6 (10 ng/ml), TNF-α (20 ng/ml), and IL-1β (1 ng/ml) for 30 min by ELISA. The values represent the mean ± SEM of 6 samples and were compared by one-way ANOVA with Newman–Keuls and Dunnett’s test for multiple comparisons. *p < 0.05, **p < 0.01, ***p < 0.001

To investigate cytokines in M1 or M2 macrophages supernatant that regulate the expression of OT and OTR, we first measured the levels of different cytokines in the supernatant. The levels of TNF-α, IL-6 and IL-1β were obviously increased in M1 supernatant (Fig. 3A–C), whereas in M2 supernatant, TGF-β levels were obviously increased (Fig. 3D). Second, we tested the effects of these cytokines on the mRNA expression of OT and OTR. Treatment with IL-6, TNF-α or IL-1β significantly decreased the mRNA expression of OT and OTR in cultured enteric neurons (Fig. 3E–G) and reduced the release of OT in cultured enteric neurons (Fig. 3J). By contrast, TGF-β increased the mRNA levels of OT and OTR in cultured enteric neurons (Fig. 3I), and IL-10 seemed to have no effect (Fig. 3H).

**Fig. 4:**
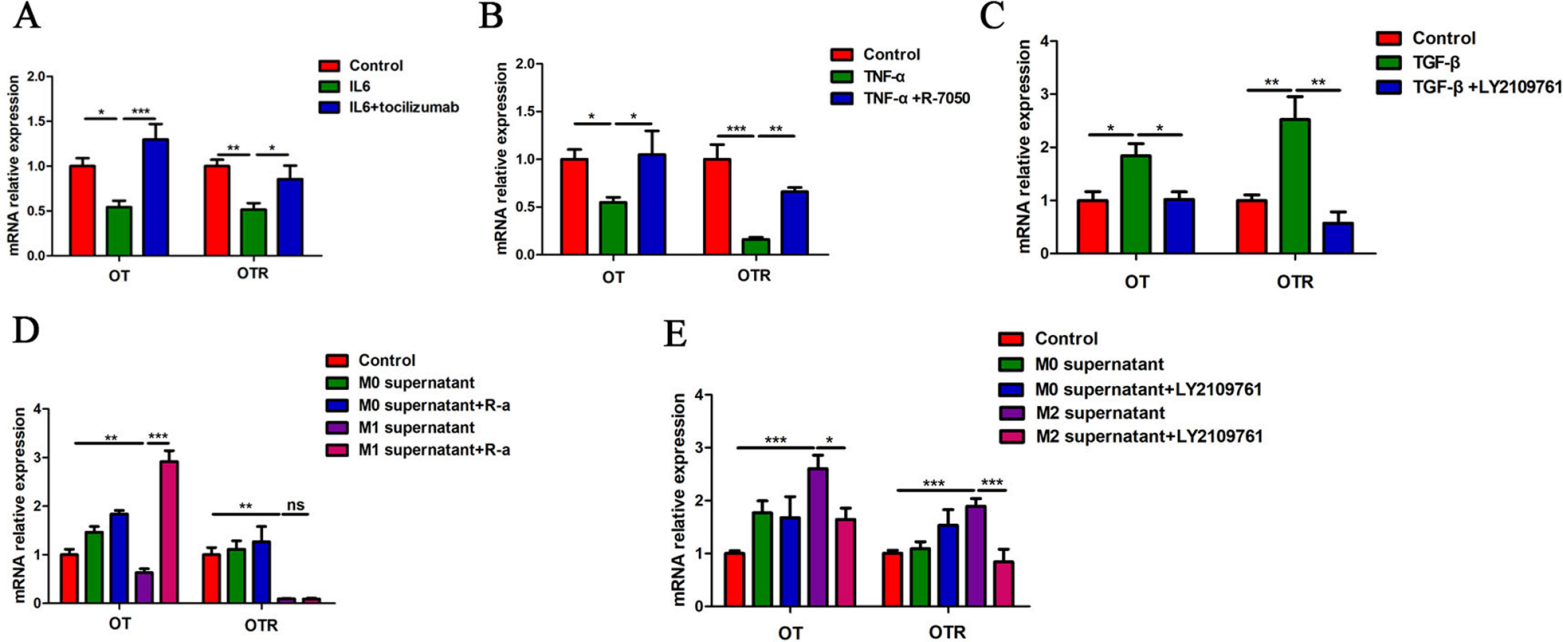
Effects of cytokine receptor antagonistson OT and OTR expression in cultured enteric neurons. **A** Cultured enteric neurons were pretreated with tocilizumab (IL-6-Ra, 1 μM) for 12 h followed by IL-6 (10 ng/ml) for 6 h. The relative mRNA expression levels of OT and OTR were analysed by qRT-PCR. **B** Cultured enteric neurons were pretreated with R-7050 (TNF-α receptor antagonist, 1 μM) for 12 h followed by TNF-α (20 ng/ml) for 6 h. The relative mRNA expression levels of OT and OTR were analysed by qRT-PCR. **C** Cultured enteric neurons were pretreated with LY2109761 (TGF-β receptor antagonist, 1 μM) for 12 h followed by TGF-β (10 ng/ml) for 6 h. The relative mRNA expression levels of OT and OTR were analysed by qRT-PCR. **D** Cultured enteric neurons were pretreated with tocilizumab, R-7050 or IL-1ra for 12 h followed by M0 and M1 supernatant for 24 h. The relative expression levels of OT and OTR mRNA were analysed by qRT-PCR. Notably, the three receptor antagonists reversed the effect of M1 supernatant on the expression of OT. **E** Cultured enteric neurons were pretreated with LY2109761 for 12 h followed by M0 or M2 supernatant for 24 h. The relative expression levels of OT and OTR mRNA were analysed by qRT-PCR. LY2109761 downregulated the mRNA expression of OT and OTR by inhibiting the expression of TGF-β. Values represent the mean ± SEM of 6 samples and were compared by one-way ANOVA with Newman–Keuls and Dunnett’s test for multiple comparisons. *p < 0.05, **p < 0.01, ***p < 0.001

To test whether the above cytokines affected the expression of OT and OTR through their receptors, we pretreated neurons with tocilizumab (IL-6 receptor antagonist), R-7050 (TNF-a receptor antagonist), or LY2109761 (TGF-β antagonist). We found that tocilizumab, R-7050 and LY2109761 reversed the inhibitory (IL-6 and TNF-α) and excitatory (TGF-β) effects of the respective agonists (Fig. 4A–C, Additional file 1: Fig. S1D). In addition, treatment with IL-1Ra, tocilizumab and R-7050 completely reversed the excitatory effect of M1 supernatant on the expression of OT, and LY2109761 completely reversed the inhibitory effect of M2 supernatant (Fig. 4D, E). Therefore, we believe that the inhibitory effect of M1 supernatant on OT and OTR expression occurs mainly through IL-1β, IL-6 and TNF-α, whereas the excitatory effect of M2 supernatant originates mainly from TGF-β.

### Inflammatory cytokines downregulate the expression of OT/OTR proteins in enteric neurons via the STAT3 or NF-κB pathway

**Fig. 5:**
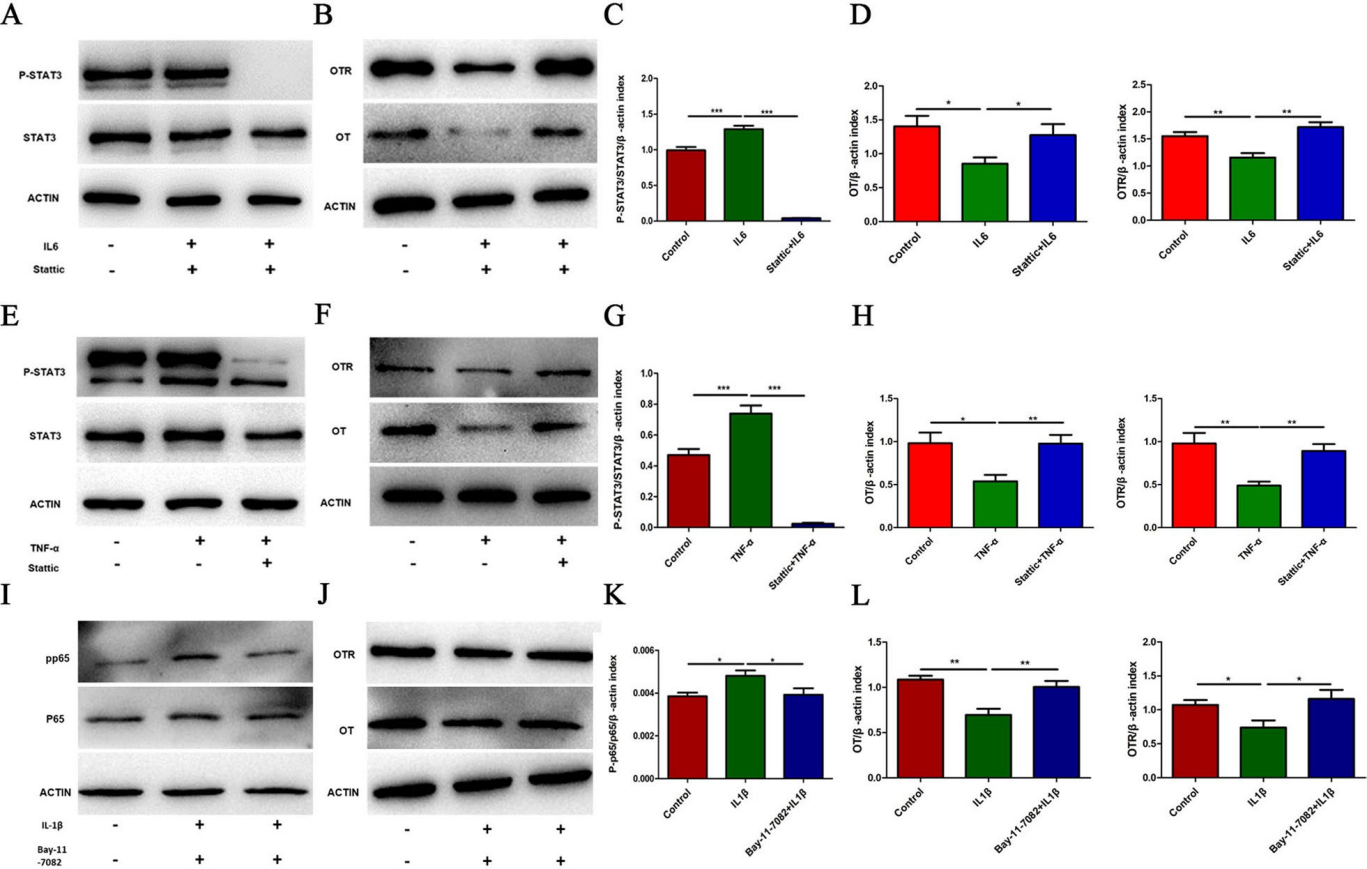
Inflammatory cytokines downregulate the protein expression of OT/OTR in enteric neurons via the STAT3 or NF-κB pathway. **A**–**D** Cultured enteric neurons were pretreated with or without Stattic (STAT3 inhibitor, 5 μM) for 12 h followed by IL6 (10 ng/ml) or TNF-α (20 ng/ml) for 24 h. The levels of phosphorylated STAT3 (p-STAT3), total STAT3, OT, and OTR were analysed by western blot, and β-actin was used to evaluate protein loading. IL-6 (10 ng/ml) or TNF-α (20 ng/ml) decreased the expression of OT and OTR by activating the STAT3 pathway. **E**, **F** Cultured enteric neurons were pretreated with or without Bay11-7082 (NF-κB inhibitor, 1 μM) for 2 h followed by IL-1β (1 ng/ml) for 24 h. The levels of phosphorylated NF-κB (p-p65), total NF-κB (p65), OT, and OTR were analysed by western blot, and β-actin was used to evaluate protein loading. IL-1β (1 ng/ml) decreased the expression of OT and OTR by activating the NF-κB pathway. Values represent the mean ± SEM of 6 samples and were compared by one-way ANOVA with Newman–Keuls for multiple comparisons. *p < 0.05, **p < 0.01, ***p < 0.001

Inflammatory factors can activate the STAT3 [30] and NF-κB pathways to participate in intracellular reactions [22, 31]. We therefore investigated whether these two pathways were involved in the downregulation of the protein expression of OT and OTR following IL-6, TNF-α and IL-1β treatment. The degree of STAT3 phosphorylation was significantly higher following treatment with IL-6 or TNF-α for 24 h than in the control group (Fig. 5A, C, E, G). In addition, the protein expression of OT and OTR decreased significantly, and this inhibitory effect was reversed by stattic (a STAT3 inhibitor, 5 μM) (Fig. 5B, D, F, H). Treatment with IL-1β did not change the degree of STAT3 phosphorylation (Additional file 1: Fig. S1F), but significantly upregulated the protein expression of p-NF-κB in enteric neurons (Fig. 5I, K). The decreases in OT and OTR expression following IL-1β administration were significantly reversed by pretreatment with Bay11-7082 (an NF-κB inhibitor, 1 μM) (Fig. 5J, L). Therefore, we believe that the inflammatory cytokines in M1 macrophages supernatant inhibit the expression of OT and OTR via different pathways. TNF-α and IL-6 may elicit their effects through STAT3 phosphorylation, whereas the influence of IL-1β is mainly related to NF-κB phosphorylation.

### The Smad2/3 pathway is involved in the upregulation of OT/OTR expression following TGF-β

**Fig. 6:**
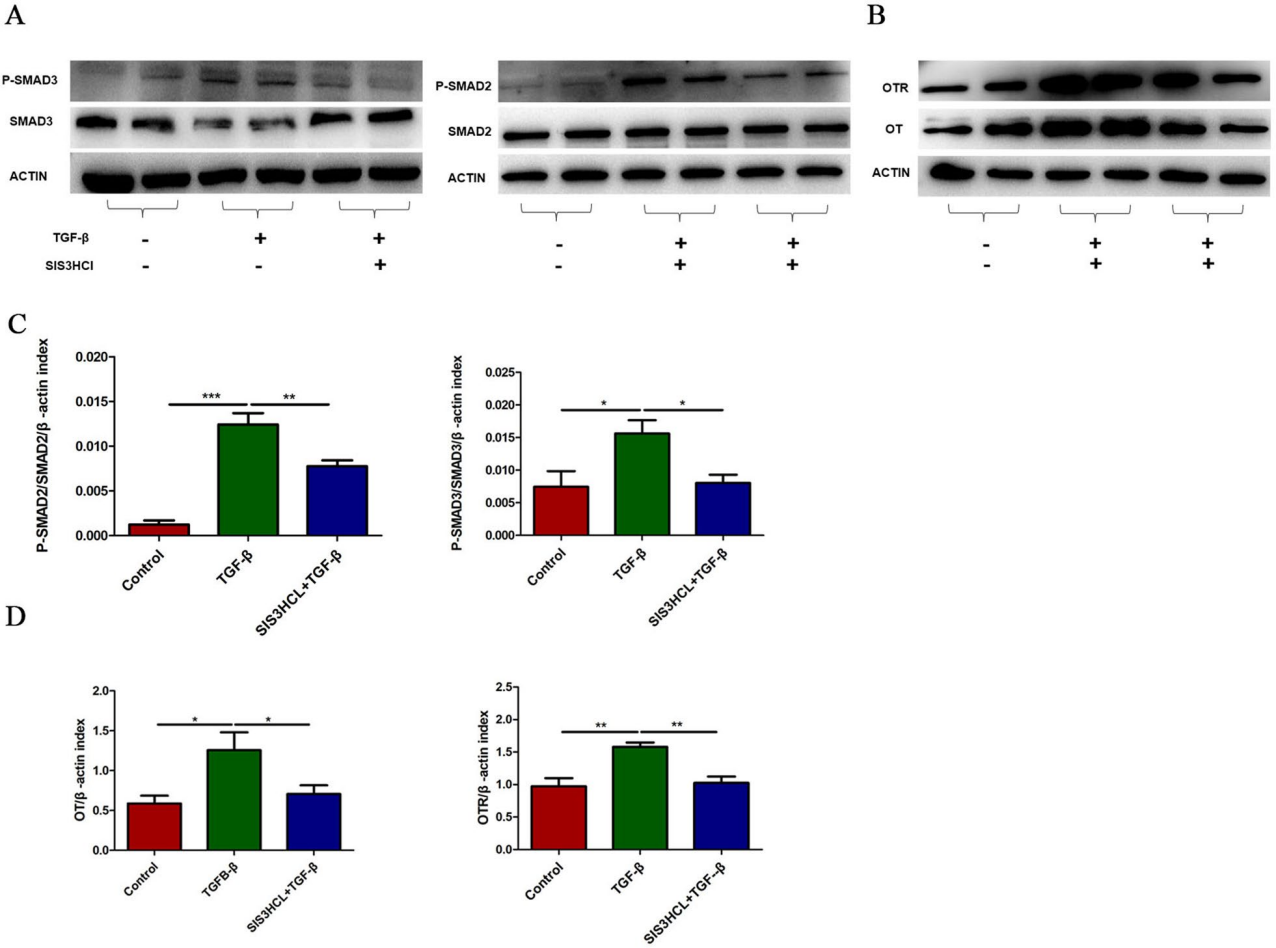
The Smad2/3 pathway is involved in the upregulation of OT/OTR expression following TGF-β treatment. **A**–**D** Cultured enteric neurons were pretreated with or without SIS3CHL (Smad3 inhibitor, 5 μM) for 12 h followed by TGF-β (10 ng/ml) for 24 h. The levels of phosphorylated Smad2 (p-Smad2), phosphorylated Smad3 (p-Smad3), total Smad2, total Smad3, OT, and OTR were analysed by western blot, and β-actin was used to evaluate protein loading. TGF-β (10 ng/ml) increased the expression of OT and OTR by activating the Smad2/3 pathway. The values represent the  mean ± SEM of 6 samples and were compared by one-way ANOVA with Newman–Keuls for multiple comparisons. *p < 0.05, **p < 0.01, ***p < 0.001

TGF-β induces a cellular response by binding transmembrane receptors [32] and phosphorylating Smad2/3 (receptor regulated Smads: R-Smads) [33]. In this study, we found that p-Smad2 and p-Smad3 expression were upregulated following TGF-β treatment for 24 h, and pretreatment with SIS3CHL (Smad3 inhibitor, 5 μM) blocked this change (Fig. 6A–D). Therefore, we believe that TGF-β increases OT and OTR expression in enteric neurons via the Smad2/3 pathway.

### Relationship between macrophage polarization and OT and OTR expression in DSS-induced colitis

**Fig. 7:**
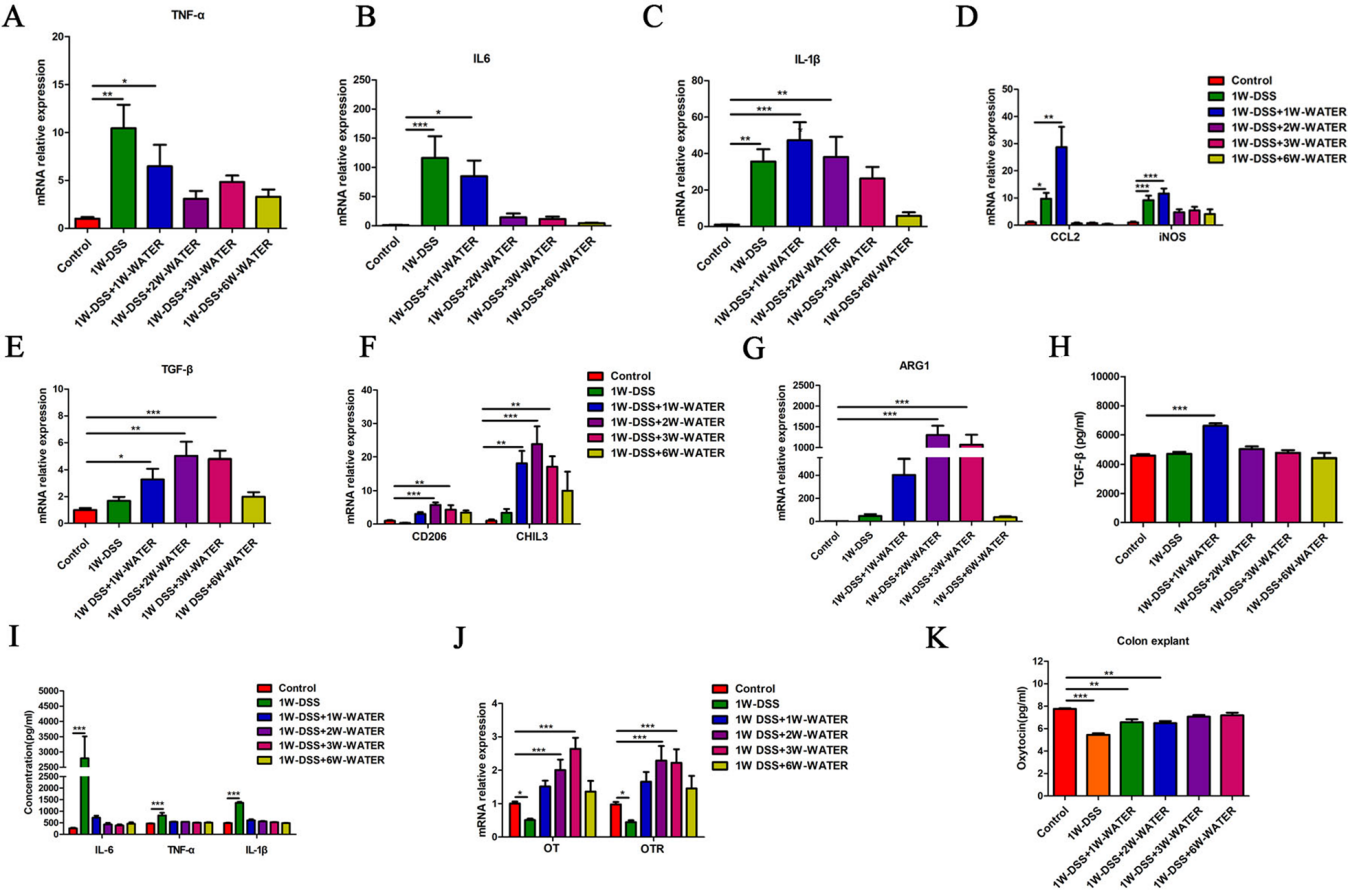
Correlation between macrophage polarization and the expression of OT and OTR in DSS-induced colitis. **A**–**D** The mRNA levels of TNF-α, IL-6, IL-1β, CCL2, and iNOS in the LMMP were measured in different periods of the DSS model by qRT-PCR. In the first and second weeks, the mRNA levels of IL-1β, IL-6, TNF-α, CCL2, and iNOS were upregulated. The above results suggested that M1 macrophages polarization. **E**–**G** The mRNA levels of TGF-β, CD206, ChIL3, and Arg1 in the LMMP were measured in different periods of the DSS model by qRT-PCR. From the third week of the model, the levels of TGF-β, CD206, ChIL3, and Arg1 mRNA were upregulated. These results suggested M2 macrophage polarization. **H**, **I** The levels of IL-6, TNF-α, IL-1β and TGF-β proteins were measured in LMMP by ELISA. **J** OT and OTR mRNA levels in LMMP were measured in different periods of the DSS model by qRT-PCR. The expression of OT and OTR decreased as inflammation occurred and increased as inflammation subsided. **K** The level of OT protein in colon explants was measured in different periods of the DSS model by ELISA. The values represent the mean ± SEM of 7 samples and were compared by one-way ANOVA with Dunnett’s test for multiple comparisons. *p < 0.05, **p < 0.01, ***p < 0.001

**Fig. 8:**
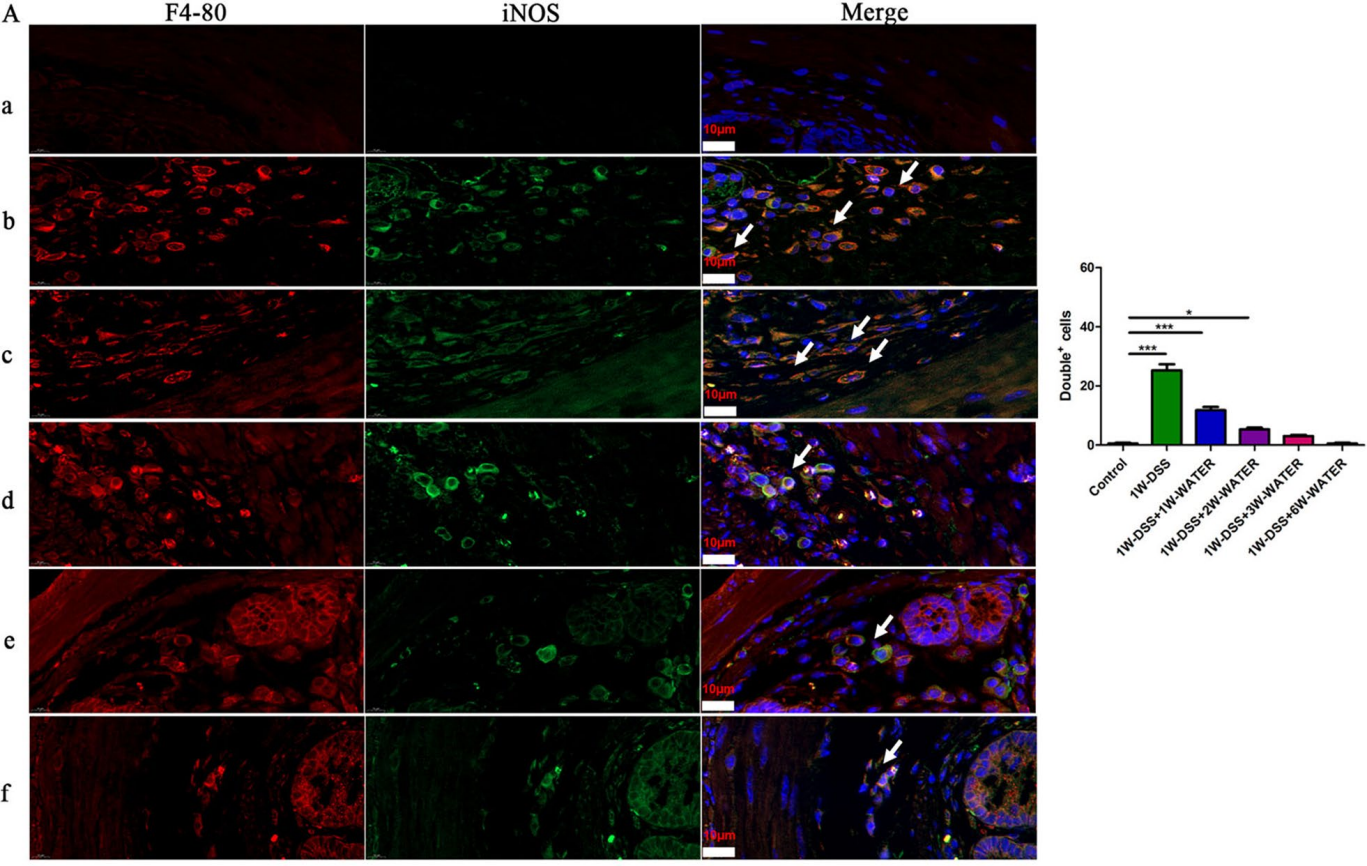
M1 macrophages are activated in the inflammatory phase. Representative photographs of double immunofluorescent staining of iNOS (green) and F4-80 (red) within randomly captured images were obtained for each section per animal. Scale bars = 10 μm. The values represent the mean ± SEM of 4 samples and were compared by one-way ANOVA with Dunnett’s test for multiple comparisons. *p < 0.05, ***p < 0.001

Our laboratory has previously shown that the OT signalling system reduces intestinal inflammation by regulating the polarization of macrophages in experimental colitis [10, 22, 34]. In this study, we explored whether the expression of the OT signalling system was impacted by DSS-induced colitis (Additional file 2: Fig. S2A). In the first 7 days during which the mice were administered DSS in drinking water, the mice in the DSS treatment group had significantly reduced body weight, reduced colon length, and a higher disease activity index than those in the control group (Additional file 2: Fig. S2B–E). These changes reached a maximum at the end of the first week after the beginning of DSS feeding and then began to decrease. At the third week, the weight of the mice and the colon length returned to normal, and the disease activity index decreased significantly (Additional file 2: Fig. S2B–E). Histological evaluation at different time periods showed that at 7 days following DSS treatment, the colon exhibited severe epithelial damage, crypt loss, submucosal oedema, and inflammatory cell infiltration. However, these histological changes began to decrease at the second week and completely disappeared at the seventh week (Additional file 2: Fig. S2F, G). Therefore, we divided DSS-induced colitis into the inflammatory phase (the first and second weeks) and the recovery phase (the third week and later) (Additional file 2: Fig. S2A). The previous data indicated that M1 supernatant, IL-1β, TNF-α, and IL-6 inhibited the mRNA expression levels of OT and OTR in cultured enteric neurons, whereas M2 supernatant and TGF-β promoted their expression (Figs. 2, 3, 4). In the inflammatory phase of DSS-induced colitis, IL-1β, TNF-α and IL-6 mRNA expression were significantly increased in the colon (Fig. 7A–C). In addition, the mRNA expression of the M1 macrophages markers CCL2 and iNOS were significantly increased (Fig. 7D). The number of M1 phenotypes (F4-80^+^/ iNOS ^+^) was significantly increased (Fig. 8). These parameters reached their highest levels at the first or second week and gradually decreased to normal in the recovery period. By contrast, the mRNA expression of TGF-β and the M2 macrophage markers CD206, Arg1, and Chil3 did not change in the first week but began to increase at the second week and reached the highest level at the third or fourth week (Fig. 7E–G). The changes in the concentrations of IL-6, IL-1β, TNF-α and TGF-β showed similar dynamics (Fig. 7H, I). Therefore, we believe that macrophages are polarized to the M1 type during the inflammatory phase and to the M2 type during the recovery phase. Next, we assessed the expression of the OT signalling system in the two phases in DSS-treated mice. The mRNA expression of OT and OTR decreased significantly during the inflammatory phase and increased significantly during the recovery phase (Fig. 7J). The level of OT in colon explant and the density of neurons in the each ganglion showed the same dynamic changes (Fig. 7K and Additional file 3: Fig. S3). We believe that the expression of the OT signalling system and macrophage polarization in the colon are correlated in these two phases of DSS-induced colitis.

### M2 macrophage polarization increases the expression of OT and OTR in the colon

To further test the hypothesis that M2 macrophage polarization increases OT and OTR expression in the gut, we used D-mannose (a commonly used glyconutrient in clinical practice) to activate macrophages [35]. The mice were fed D-mannose for 7 days. The body weight of the mice decreased on the second day and began to recover on the fourth day (Additional file 4: Fig. S4A). There were no changes in eating habits and the histological structure of the colon (Additional file 4: Fig. S4B, C), but the size and weight of the caecum and colon increased (Additional file 4: Fig. S4D). We also confirmed those results in female mice (Additional file 5: Fig. S5A). D-Mannose has been reported to inhibit M1 polarization [35]. After 7 days of D-mannose feeding, iNOS mRNA expression in the colon was decreased, but the mRNA expression of IL-6, IL-1β and TNF-α did not change (Additional file 4: Fig. S4F). Surprisingly, the mRNA expression of surface markers of M2 macrophages in the colon, including CD206, Arg1, Ym1 and Chil3, significantly increased, and the anti-inflammatory factors TGF-β and IL10 were also significantly upregulated (Additional file 4: Fig. S4E). Therefore, we believe that D-mannose promotes the polarization of M2 macrophages and inhibits the polarization of M1 macrophages. Consistent with the change in macrophage polarization, OT and OTR expression were also significantly upregulated (Additional file 4: Fig. S4G). We also confirmed those results in female mice (Additional file 5: Fig. S5B).

**Fig. 9:**
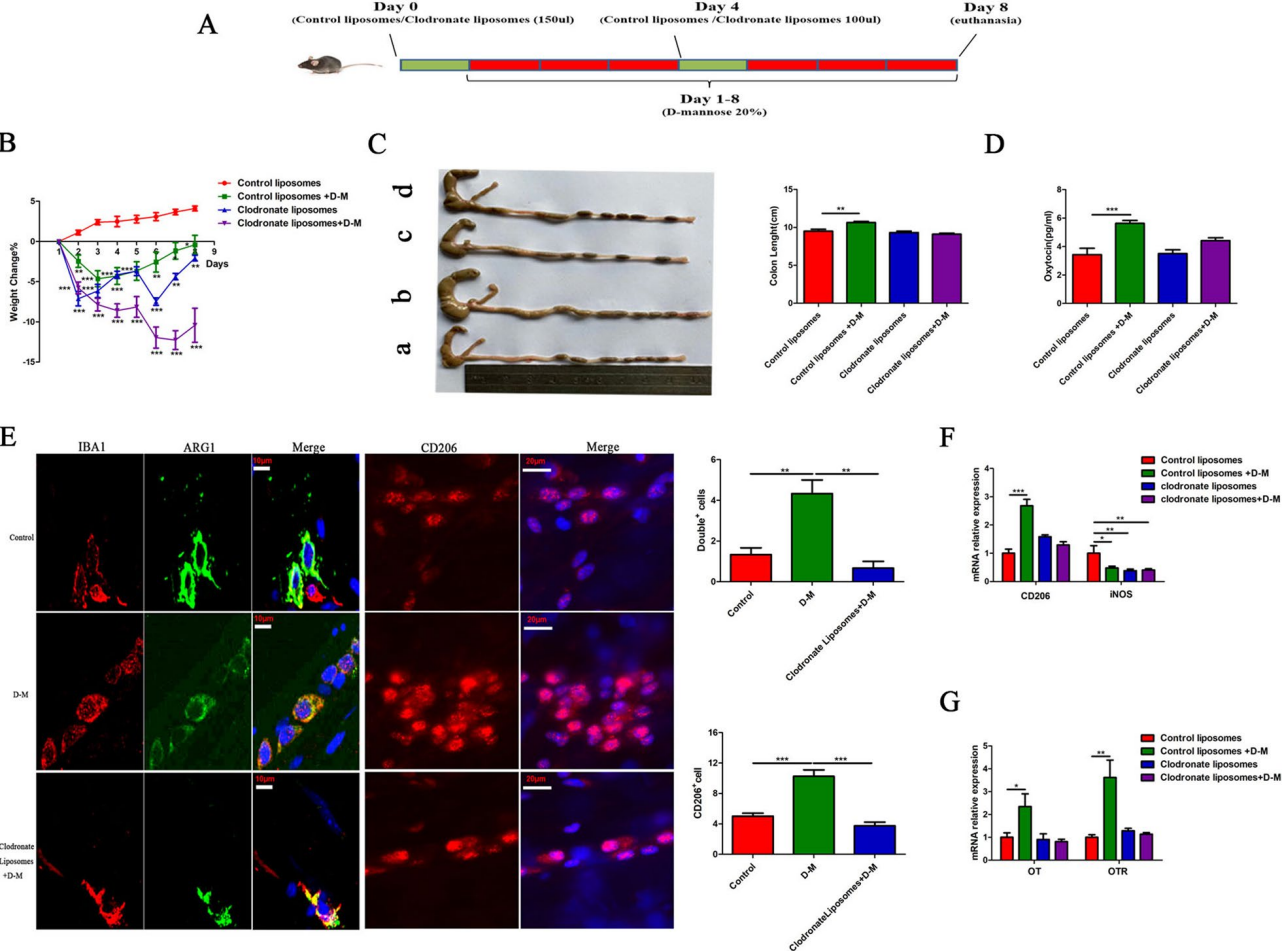
M2 macrophage polarization increases the expression of OT and OTR in the colon. **A** Animal pattern. The day before and on the fourth day of the D-mannose model, clodronate liposomes/control liposomes (200 µL/mouse) were injected intraperitoneally, and 20% D-mannose was fed daily in drinking water for 7 consecutive days. **B** The body weight change during the 7-day D-mannose model. **C** Statistical comparison of colon lengths in the different groups. **D** The concentration of OT in plasma after injecting control liposomes and clodronate liposomes was measured by ELISA. **E** Muscularis macrophages in colon cross sections (left) and whole mount myenteric plexus (right) stained for anti-Iba1 (left) or anti-Arg1 antibodies (left) and CD206 (right). Scale bars, 10 μm for cross sections; 20 μm for whole mount images. Images are representative of 2 independent experiments. N = 4/group. **F** The levels of CD206 and iNOS mRNA after injecting control liposomes and clodronate liposomes were measured by qRT-PCR. **G** The levels of OT and OTR mRNA of LMMP after injecting control liposomes and clodronate liposomes were measured by qRT-PCR. Notably, after macrophages were depleted, the expression of OT and OTR decreased significantly. The values represent the mean ± SEM of 6 samples and were compared by one-way ANOVA with Dunnett’s test for multiple comparisons. *p < 0.05, **p < 0.01, ***p < 0.001

To test the hypothesis that the increase in the OT signalling system following D-mannose treatment is attributable to M2 polarization, we depleted macrophages via systemic administration of clodronate liposomes [29, 36–38] (Fig. 9A) and observed the expression of the OT signalling system. After clodronate liposome pretreatment, the number of Iba1/Arg1 double-positive cells (Fig. 9E, left), CD206-positive cells (Fig. 9E, right) and the expression of M1 and M2 macrophage surface markers (Fig. 9F) were significantly reduced. Treatment with clodronate liposomes decreased body weight, but did not influence the eating and drinking habits of the mice nor the size and weight of the colon and caecum (Fig. 9B, C and Additional file 4: Fig. S4I, J). Pretreatment with clodronate liposomes significantly reversed the upregulation of OT and OTR expression in the colon induced by systemic administration of D-mannose (Fig. 9D, G). On the other hand, D-mannose itself did not affect the expression of OT and OTR mRNA in the cultured enteric neurons (Additional file 4: Fig. S4H). These data further prove our hypothesis that M2 macrophages promote the expression of the OT signalling system in the colon.

### *Peg3* is involved in the upregulation of OT and OTR expression induced by M2 macrophage polarization

**Fig. 10:**
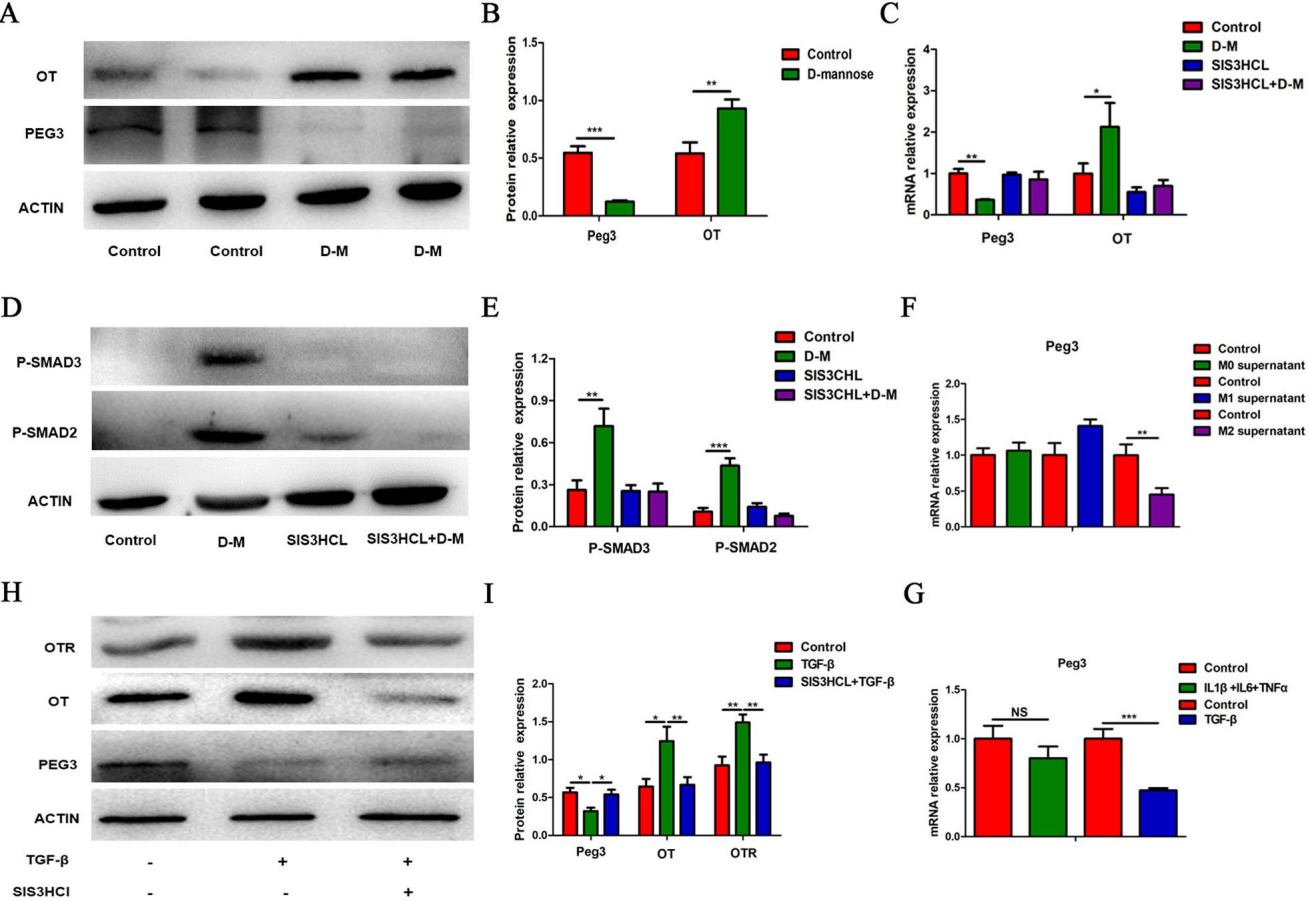
*Peg3* is involved in the upregulation of OT and OTR expression induced by M2 macrophage polarization. **A**, **B** The expression of *Peg3* and OT of LMMP in the D-mannose model was detected by western blot analysis. **C** The levels of *Peg3* and OT mRNA of LMMP in the D-mannose model after injection of SIS3HCL (Smad3 inhibitor, 2.5 μg/g) were measured by qRT-PCR. **D**, **E** Representative immunoblots for the detection of phosphorylated Smad2/3 after SIS3HCL injection by western blot analysis. β-actin was used to evaluate protein loading. **F** The level of *Peg3* mRNA in cultured enteric neurons treated with or without conditioned medium was measured by qRT-PCR. M2 supernatant suppressed the expression of *Peg3*. **G** The level of *Peg3* mRNA in cultured enteric neurons treated with or without IL-1β (1 ng/ml), IL-6 (10 ng/ml), TNF-α (20 ng/ml) or TGF-β (10 ng/ml) was measured by qRT-PCR. **H**, **I** Cultured enteric neurons were pretreated with SIS3CHL (Smad3 inhibitor, 5 μM) for 12 h followed by TGF-β (10 ng/ml) for 24 h. Only TGF-β inhibited the expression of *Peg3*. **I** The protein levels of *Peg3*, OT, and OTR were analysed by western blot, and β-actin was used to evaluate protein loading. The values represent the mean ± SEM of 6 samples and were compared by *t-*test or one-way ANOVA with Dunnett’s test for multiple comparisons. *p < 0.05, **p < 0.01, ***p < 0.001

*Peg3* (Paternally Expressed Gene 3) encodes a DNA-binding protein that acts as an inhibitory transcriptional regulator of the expression of OTR [39]. Therefore, we hypothesized that cytokines released from polarized macrophages might influence OT signalling system expression through *Peg3*. To prove this hypothesis, we first tested the expression of *Peg3* and OT in D-mannose-treated mice. Seven days following D-mannose treatment, the expression of *Peg3* was inhibited, whereas the expression of OT was increased (Additional file 4: Fig. S4K and Fig. 10A, B). Because TGF-β promotes the expression of the OT signalling system through the Smad2/3 signalling pathway, we investigated whether the downregulation of *Peg3* expression following D-mannose treatment also occurred through Smad2/3. We found that injection of SIS3CHL, a Smad3 blocker, significantly abolished the effect of D-mannose on the expression of *Peg3* (Fig. 10C–E). We repeated this experiment in cultured enteric neurons and found that treatment with M0 and M1 supernatant and inflammatory cytokines, including IL-6, IL-1β and TNF-α, did not change the expression level of *Peg3*. By contrast, treatment with M2 supernatant and TGF-β significantly reduced *Peg3* expression compared with the control group of cultured enteric neurons (Fig. 10F, G). Blocking the Smad3 signalling pathway with SIS3HCI significantly attenuated the downregulation of *Peg3* expression and upregulation of OT and OTR expression (Fig. 10H, I). These results indicate that M2 macrophages and TGF-β increase OT and OTR expression through downregulation of *Peg3* expression.

## Discussion

**Fig. 11:**
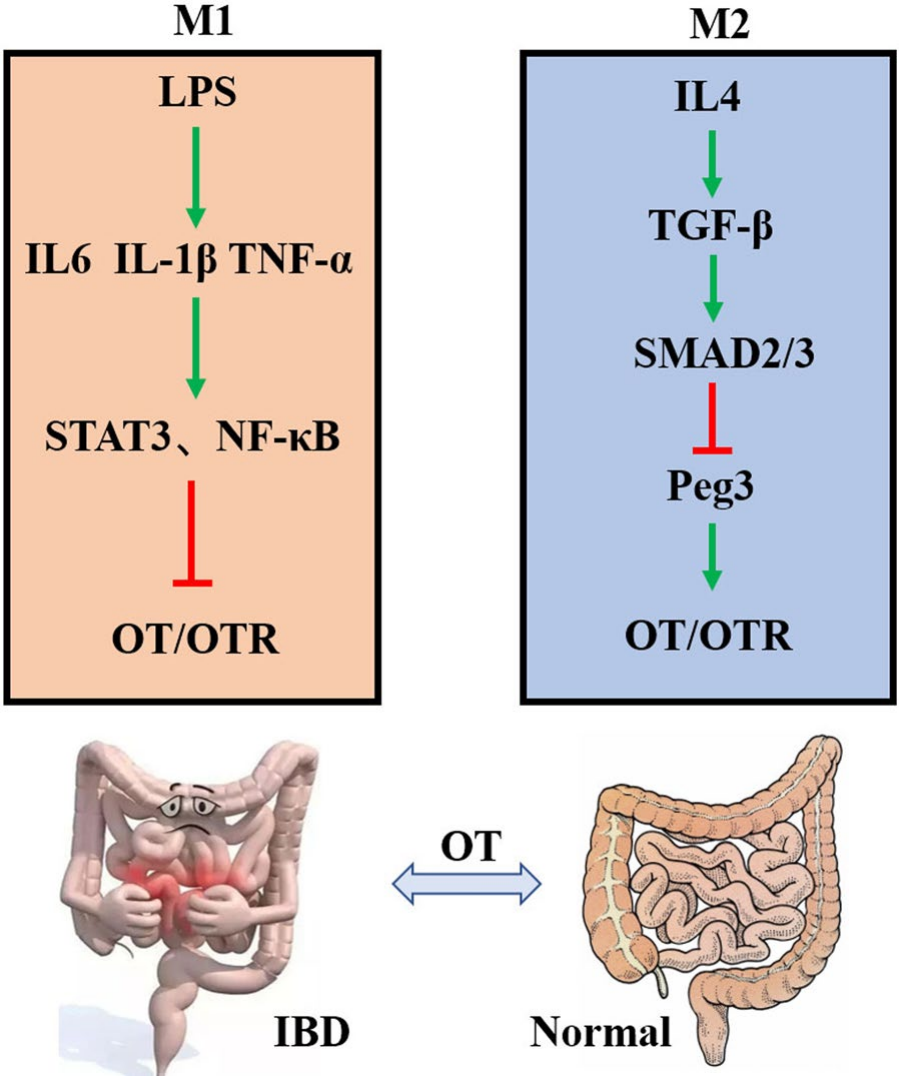
The mechanism by which macrophage polarization modulates the OT signalling system. LPS induces M1-like polarization to secrete proinflammatory factors that activate the STAT3 and NF-κB pathways to inhibit the OT signalling system in enteric neurons. IL4 induces M2-like polarization to secrete anti-inflammatory factors that activate the Smad2/3 pathway to upregulate the OT signalling system by inhibiting the expression of *Peg3* in enteric neurons. OT may be a potent agent for the treatment of gastrointestinal disorders caused by immune dysfunction

Our current study demonstrates that macrophage polarization affects the expression of the OT signalling system in the gut. In vitro, M1 macrophages inhibit the expression of the OT signalling system through secretion of proinflammatory factors, including IL-1β, IL-6 and TNF-α, and the intracellular STAT3 and NF-κB pathways in enteric neurons. On the other hand, M2 macrophages promote the expression of the OT signalling system in enteric neurons through secretion of TGF-β and the intracellular Smad2/3-*Peg3* pathway. This report is the first to demonstrate that macrophage polarization differentially regulates OT and OTR expression in enteric neurons (Fig. 11).

Levels of the stress hormone OT change during infection and inflammation [40–42]. In response to LPS stimulation, recruited M1 macrophages produce large amounts of proinflammatory cytokines, such as IL-1β, IL-6, and TNF-α. IL-4 treatment induces M2 macrophage polarization and the release of anti-inflammatory cytokines such as TGF-β and IL-10 [43]. We found that proinflammatory cytokines, including IL-1β, IL-6 and TNF-α, inhibited OT and OTR expression in enteric neurons, whereas TGF-β had an excitatory effect. These data consistent with the observation that M1 supernatant inhibits OT and OTR expression and that M2 supernatant increases OT and OTR expression. This result was also verified in enteric neurons extracted from female mice. We therefore concluded that the cytokines in the M1 supernatant that inhibit OT and OTR expression in enteric neurons might be IL-1β, IL-6 and TNF-α, while the cytokines in M2 supernatant that increase OT and OTR expression might be TGF-β. Pretreatment with receptor antagonists of these cytokines abolished the effect of M1 and M2 supernatant. Therefore, we believe that following activation, M1 macrophages decrease OT and OTR expression in enteric neurons through the proinflammatory cytokines IL-1β, IL-6 and TNF-α, while M2 macrophages increase OT and OTR expression through the anti-inflammatory cytokine TGF-β. After these inflammatory factors bind to their receptors, they participate in biological processes through a series of signalling pathways, such as JAK-STAT3 and NF-κB [30, 31, 44, 45]. In agreement with the above observations, we observed that the proinflammatory factors IL-6 and TNF-α activate the STAT3 signalling pathway and that IL-1β activates the NF-κB signalling pathway to inhibit the expression of OT and OTR. Data from experiments in which the STAT3/NF-κB signalling pathway was blocked further support these results. One of the interesting findings of the present study is that M2-polarized macrophages increase the expression of OT and OTR in enteric neurons, which might be mediated by TGF-β. TGF-β mainly regulates downstream cell responses through Smad and non-Smad signalling pathways [33, 46]. Our experiments confirmed that TGF-β activates Smad2/3 and promotes the expression of OT and OTR. Both LY2109761 (TGF-β receptor antagonist) and SIS3HCL (Smad3 blocker) completely abolished the excitatory effect of TGF-β on OT and OTR. Therefore, we believe that TGF-β upregulates the expression of OT and OTR in enteric neurons mainly through the intracellular Smad2/3 pathway.

To confirm the correlation between macrophage polarization and OT/OTR expression in vivo, we used a DSS-induced colitis model and investigated the temporal relationship between inflammation and OT/OTR expression. The disease activity index and the levels of proinflammatory cytokines in the colon increased and reached the highest level at the second week, so we defined this period as the inflammatory phase. In this period, the activation of M1 macrophages and the increase in proinflammatory cytokines were correlated with decreased OT and OTR expression in the ENS. Three weeks after DSS administration, the disease activity of the mice gradually returned to normal, and the expression of M2 macrophages markers and anti-inflammatory cytokines began to increase, reaching their highest level at the fourth week and returning to normal at the fifth to seventh weeks. We defined this period as the recovery period. In this period, macrophage polarization to the M2 type and TGF-β release were correlated with increased OT/OTR expression in the ENS. This observation further supports our hypothesis that following acute inflammation, dynamic changes in OT/OTR expression are correlated with differences in macrophage polarization in the gut. M1 macrophage polarization is correlated with decreased OT/OTR expression in the inflammatory period, whereas M2 macrophage polarization is correlated with increased OT/OTR expression in the recovery period. To the best of our knowledge, this study is the first to examine the dynamic changes in OT/OTR expression in the ENS following DSS-induced colitis. Although we found that OT inhibited the polarization of macrophages to the M1 type and promoted polarization to the M2 type and that OTR-deficient mice were more susceptible to DSS-induced colitis [22, 42], the anti-inflammatory effect of OT might not be very important during the acute inflammatory period. During this period, the release of proinflammatory cytokines from M1 macrophages was associated with decreased expression of OT and OTR in the ENS. However, interestingly, OT/OTR expression increased during the recovery period, possibly due to polarization of M2 macrophages and the release of TGF-β. During the recovery period of acute inflammation, the polarization of M2 macrophages and the release of anti-inflammatory cytokines are primarily responsible for confining inflammation and promoting the proliferation of damaged tissue [14, 47–50]. During this period, there was positive feedback between M2 macrophage activity and the expression of OT and OTR in the ENS, so it is possible that the OT signalling system in the ENS is involved in tissue repair following local acute inflammation in the gut.

D-Mannose prevents acute lung injury by regulating PPAR-γ and TGF-β levels [25] and inhibits LPS-induced macrophage activation by impairing IL-1β production. In vivo, D-mannose improves endotoxaemia and relieves colitis [35]. Therefore, we used D-mannose to induce polarization of M2 macrophages in mice. Following D-mannose administration, the expression of iNOS in the gut was significantly downregulated, whereas TGF-β, CD206, and Arg1 expression were significantly increased. OT and OTR expression in the colon were also increased. The above data indicate that D-mannose inhibits the polarization of M1 macrophages and promotes the polarization of M2 macrophages. The increases in OT and OTR expression following treatment with D-mannose might be due to polarization of M2 macrophages. We also confirmed those results in female mice. To further test this hypothesis, we used clodronate liposomes to deplete macrophages in vivo. The upregulation of OT and OTR expression was abolished by the depletion of macrophages. Therefore, we believe that, in vivo, D-mannose increases OT and OTR expression in the ENS through polarization of macrophages to the M2 type. Another interesting finding in these experiments was that following D-mannose administration, the size and weight of the gut were increased, although the histological structure of the intestine did not change. These changes were macrophages dependent. Therefore, we concluded that these changes may have been caused by the release of cytokines such as TGF-β from polarized M2 macrophages and OT released from the ENS.

*Peg3* is widely expressed in various human and mouse tissues and encodes a DNA-binding protein that is involved in the control of specific target genes with different cell functions [51]. *Peg3* also functions as a transcriptional regulator of the expression of OTR. In *Peg3*-KO mice, the expression of OTR in the hypothalamus and breast is upregulated [39]. In this study, we first proved that M2 supernatant and TGF-β promoted the expression of OT by suppressing the expression of *Peg3* in enteric neurons. However, M0/M1 macrophages and proinflammatory factors did not affect the expression of *Peg3*.

In recent years, studies have shown that OT has anti-inflammatory effects in addition to its effects on behaviour, lactation and childbirth [1, 52]. OT acts as a neuropeptide to inhibit atherosclerosis and reduce inflammation of the visceral fat depot [53, 54]. The administration of exogenous oxytocin improves wound healing by reducing the concentration of cortisol [55]. Studies have also pointed out that OT improves the antioxidant status of colon tissue and improves colon damage [7]. We previously found that OT relieves colitis by promoting the polarization of anti-inflammatory macrophages [22]. Our results indicate that OT is likely an important signalling molecule between neurons and macrophages and may be a potential drug target for the treatment of intestinal diseases caused by immune abnormalities.

Many reports have indicated that the ENS cooperates with intestinal macrophages to maintain intestinal immune homeostasis [56, 57]. The unique colocalization of  MMs and enteric neurons has attracted great attention from researchers [58]. Emerging data suggest that MMs secrete cytokines to protect nerves from damage and promote nerve regeneration [59]. The secretion of these cytokines might be the result of the interaction between the ENS and the immune system. In this study, we found that macrophage polarization affected the expression of the enteric neuron OT signalling system. In a recent study, we reported that the OT signalling system regulates macrophage polarization. The OT signalling system in the ENS is very likely to be the signalling molecule for the dialogue between intestinal macrophages and neurons. This ‘‘feedback loop’’ between macrophages and enteric neurons could be a pivotal ‘‘controller’’ that determines the tendency toward homeostasis or disease.

## Conclusion

In summary, the present findings demonstrate that macrophage polarization affects the expression of OT signalling system in the gut. The OT signalling system may also be a potential drug target for the treatment of intestinal diseases caused by abnormal immune systems.

## Supplementary Information

**Additional** **file** **1.** **Fig.** **S1.** Effects of different drugs on the expression of OT signalling system in enteric neurons and IL1β does not affect the STAT3 pathway. (A, B) LPS (100 ng/ml) or IL-4 (10 ng/ml) stimulated cultured enteric neurons for 24 h did not affect levels of OT and OTR mRNA. (C) The secretion of OT was detected in cultured enteric neurons by ELISA. (N=4). (D) The effect of TGF-β receptor inhibitor (LY2109761) on OT was measured by western blot. (E) To exclude the influence of the menstrual cycle, we extracted enteric neurons from bilateral ovariectomized (OVX) female mice one week after surgery [1]. The level of OT mRNA in cultured enteric neurons in female mice was detected with or without conditioned medium treatment for 24 h via qRT-PCR. (F) The activation of IL-1β on STAT3 signalling pathway was detected by western blot. The values represent the mean ± SEM of 6 samples and were compared by *t*-test or one-way ANOVA with Newman–Keuls  for multiple comparisons. *p < 0.05, *** *p*<0.001 vs. control group.

**Additional file 2.** **Fig.** **S2.** Dynamic changes of various parameters of DSS-induced enteritis model over time. (A)Schematic diagram of mouse colitis model (Inflammation and recovery periods). The mice drank 2.5% DSS for 7 consecutive days, and drank water from the second week. (B) Body weight changed of WT mice in different periods of the DSS model. (C, D) The lengths of the colon from different groups were statistically compared. (E) Representative disease activity index was counted in each group based on body weight loss, stool consistence and hematochezia. (F) Representative H&E staining colonic section in each group (Scale bar: 100μm). (G) Histological assessment of the indicated group. The values represent the mean ± SEM of 7 samples and were compared by *t*-test or one-way ANOVA with Dunnett’s testfor multiple comparisons. *p < 0.05, *** *p*<0.001 vs. control group.

**Additional file 3.** **Fig.** **S3.** The changes of neuron density in each ganglion at different periods of the DSS model. Representative photographs of immunofluorescent staining of tubulin (green) within randomly captured images were obtained for each section per animal. (Scale bar: 10μm). The values represent the mean ± SEM of 4 samples and were compared by one-way ANOVA with Dunnett’s test for multiple comparisons. ** *p*<0.01 vs. control group.

**Additional file 4.** **Fig.** **S4.** D-Mannose promoted polarization of M2 macrophages and inhibited the polarization of M1 macrophages. (A, B) Changes in weight and eating habits in D-mannose model. (C) Representative H&E staining colonic section in each group (Scale bar: 100μm). (D) The lengths of the colon from different groups were statistically compared. (E) The levels of CD206, Arg1, TGF-β, IL10, Chil3, YM1 mRNA of LMMP induced by D-mannose for 7 days were measured by qRT-PCR. (F) The levels of iNOS, IL-1β, IL-6, TNF-α mRNA of LMMP induced by D-mannose for 7 days were measured by qRT-PCR. (G) The levels of OT and OTR mRNA of LMMP induced by D-mannose for 7 days were measured by qRT-PCR. (H) The levels of OT and OTR mRNA by D-mannose for 24 h in enteric neurons were measured by qRT-PCR. (I) Eating habits of mice had no change during the 7 days D-mannose model. (J) The level of *Peg3* mRNA of LMMP in D-mannose model was measured by qRT-PCR. The expression of *Peg3* of LMMP in the D-mannose group was significantly down-regulated compared with the control group (K) The level of *Peg3* mRNA of LMMP in D-mannose model was measured by qRT-PCR. The expression of *Peg3* of LMMP in the D-mannose group was significantly down-regulated compared with the control group. The values represent the mean ± SEM of 6 samples and were compared by *t*-test for multiple comparisons. *p < 0.05, ** *p*<0.01, *** *p*<0.001 vs. control group.

**Additional file 5.** **Fig.** **S5.** The expression of OT was significantly increased in OVX-female mice after drinking D-mannose for 7 days. (A) The change of colon length in female mice. (B) The level of OT mRNA was detected by QPCR in OVX-female mice after drinking D-mannose for 7 days. The values represent the mean ± SEM of 6 samples and were compared by *t*-test for multiple comparisons. **p<0.01, *** *p*<0.001.

## Data Availability

The data generated during our study cannot be made publicly available due to the data safety concern. But the data are available from the corresponding author on reasonable request.

## Abbreviations

ENS: Enteric nervous system
LMMP: Longitudinal muscle myenteric plexus
OT: Oxytocin
OTR: Oxytocin receptor
DAPI: 4',6' Diamidino-2-phenylindole dihydrochloride hydrate
FBS: Foetal bovine serum
IHC: Immunohistochemistry
LPS: Lipopolysaccharide
qRT-PCR: Reverse transcriptase quantitative real-time PCR
MMs: Muscularis macrophages
IL: Interleukin
TNF-α: Tumour necrosis factor-α
TGF-β: Transforming growth factor β
Arg1: Arginase1
STAT3: Signal transducer and activator of transcription3
Chil3: Chitinase-like 3
DSS: Dextran sulfate sodium
D-M: D-mannose
IBD: Inflammatory bowel disease
iNOS: Inducible nitric oxide synthase
