# Supplementary figures and images for: The effect of macrophage polarization on the expression of the oxytocin signalling system in enteric neurons
Source: J Neuroinflammation. 2021 Nov 8;18:261. doi: 10.1186/s12974-021-02313-w (PMC8573870; doi:10.1186/s12974-021-02313-w)

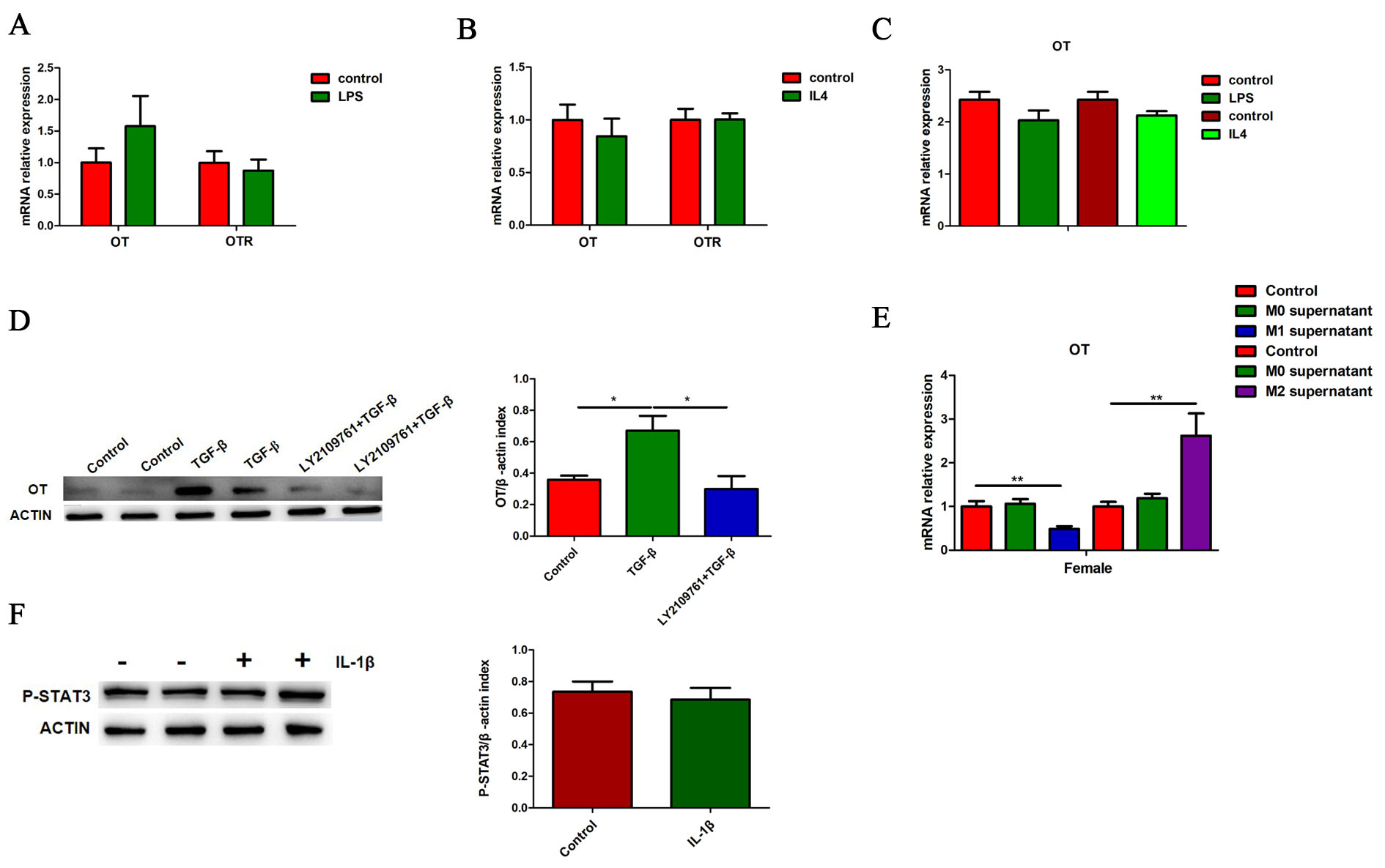

Supplement: Supplementary file 1 — Additional file 1. Fig. S1. Effects of different drugs on the expression of OT signalling system in enteric neurons and IL1β does not affect the STAT3 pathway. (A, B) LPS (100 ng/ml) or IL-4 (10 ng/ml) stimulated cultured enteric neurons for 24 h did not affect levels of OT and OTR mRNA. (C) The secretion of OT was detected in cultured enteric neurons by ELISA. (N=4). (D) The effect of TGF-β receptor inhibitor (LY2109761) on OT was measured by western blot. (E) To exclude the influence of the menstrual cycle, we extracted enteric neurons from bilateral ovariectomized (OVX) female mice one week after surgery [1]. The level of OT mRNA in cultured enteric neurons in female mice was detected with or without conditioned medium treatment for 24 h via qRT-PCR. (F) The activation of IL-1β on STAT3 signalling pathway was detected by western blot. The values represent the mean ± SEM of 6 samples and were compared by t-test or one-way ANOVA with Newman–Keuls for multiple comparisons. *p < 0.05, *** p<0.001 vs. control group. [file 12974_2021_2313_MOESM1_ESM.jpg]

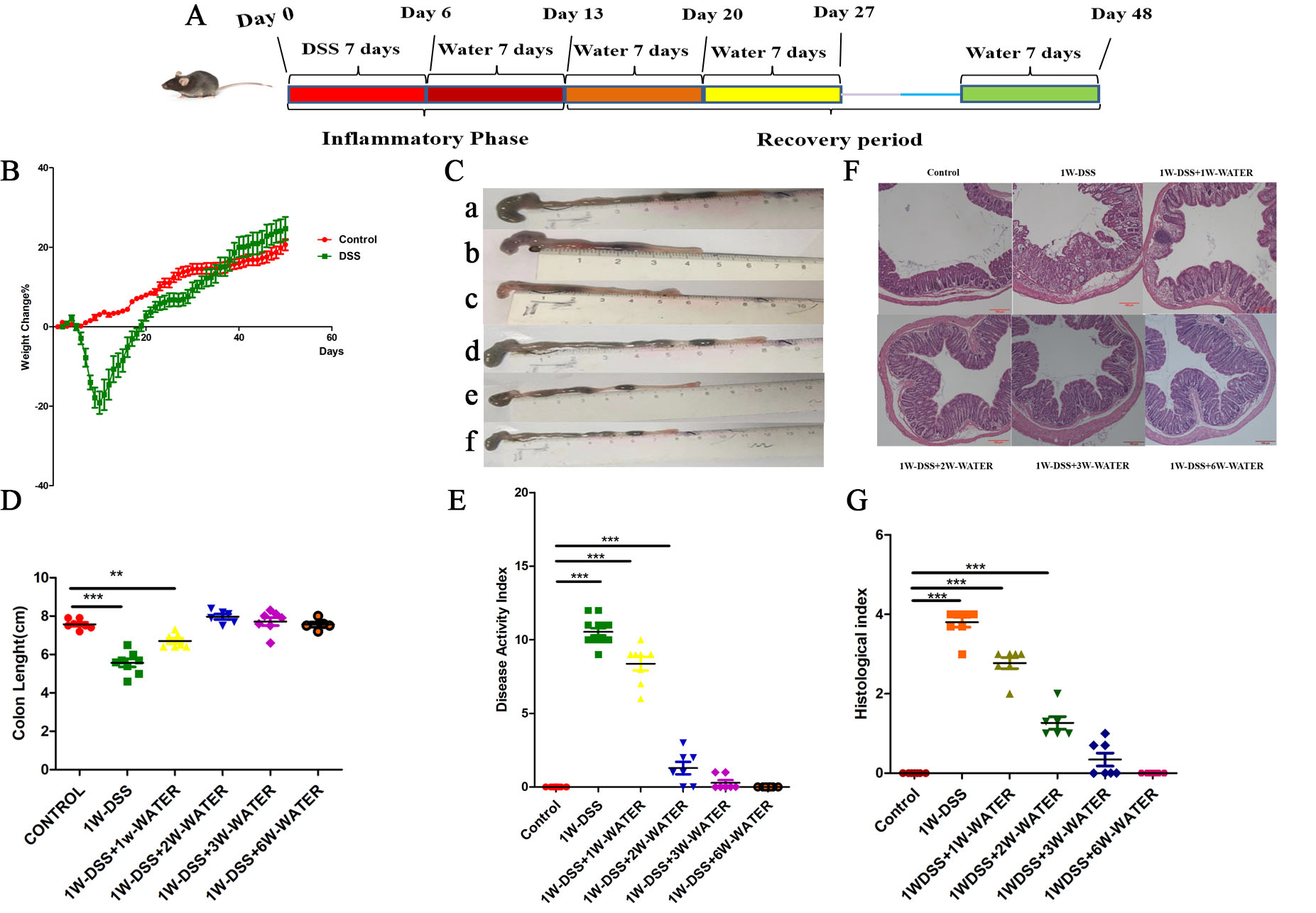

Supplement: Supplementary file 2 — Additional file 2. Fig. S2. Dynamic changes of various parameters of DSS-induced enteritis model over time. (A)Schematic diagram of mouse colitis model (Inflammation and recovery periods). The mice drank 2.5% DSS for 7 consecutive days, and drank water from the second week. (B) Body weight changed of WT mice in different periods of the DSS model. (C, D) The lengths of the colon from different groups were statistically compared. (E) Representative disease activity index was counted in each group based on body weight loss, stool consistence and hematochezia. (F) Representative H&E staining colonic section in each group (Scale bar: 100μm). (G) Histological assessment of the indicated group. The values represent the mean ± SEM of 7 samples and were compared by t-test or one-way ANOVA with Dunnett’s testfor multiple comparisons. *p < 0.05, *** p<0.001 vs. control group. [file 12974_2021_2313_MOESM2_ESM.jpg]

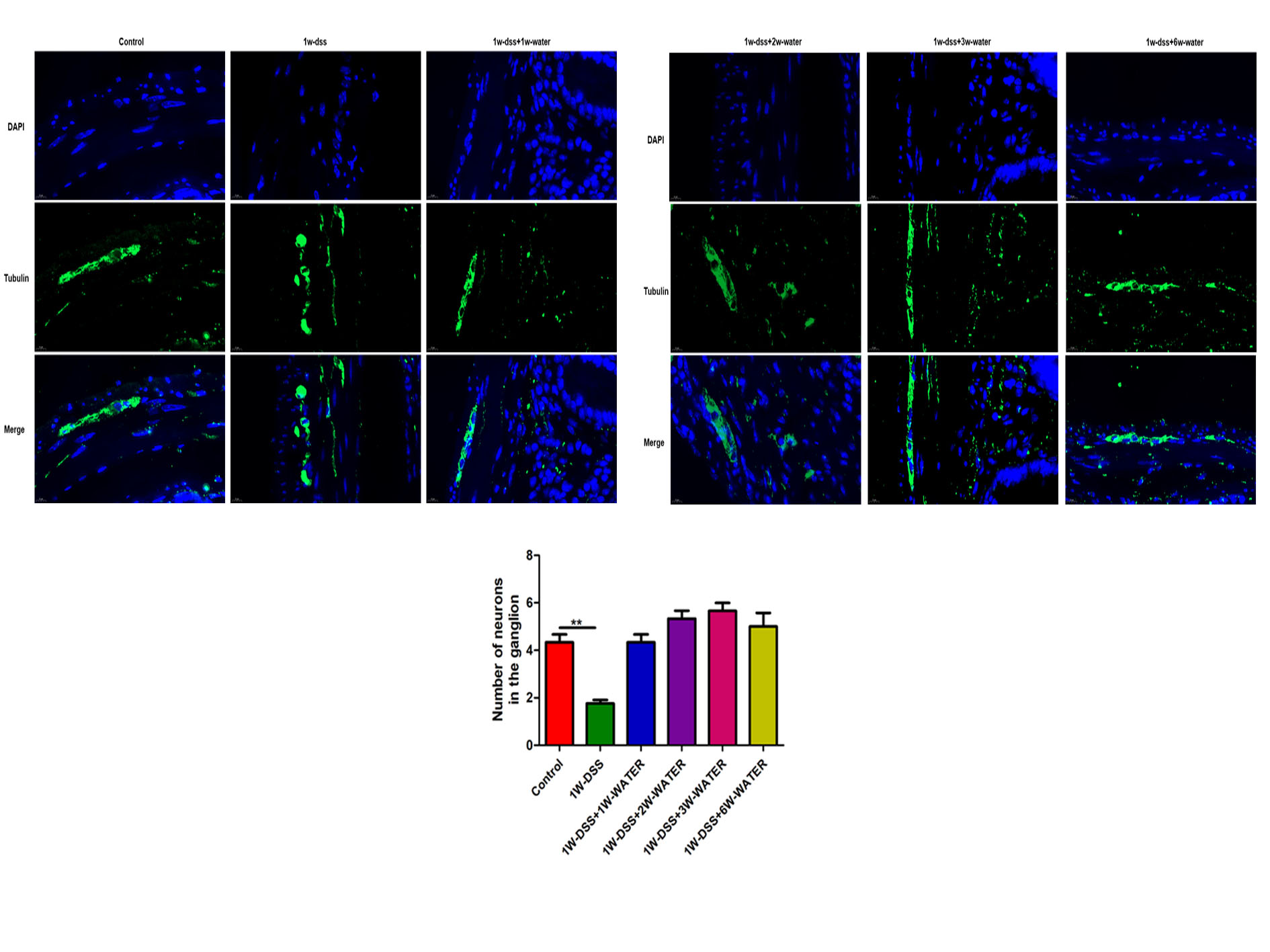

Supplement: Supplementary file 3 — Additional file 3. Fig. S3. The changes of neuron density in each ganglion at different periods of the DSS model. Representative photographs of immunofluorescent staining of tubulin (green) within randomly captured images were obtained for each section per animal. (Scale bar: 10μm). The values represent the mean ± SEM of 4 samples and were compared by one-way ANOVA with Dunnett’s test for multiple comparisons. ** p<0.01 vs. control group. [file 12974_2021_2313_MOESM3_ESM.jpg]

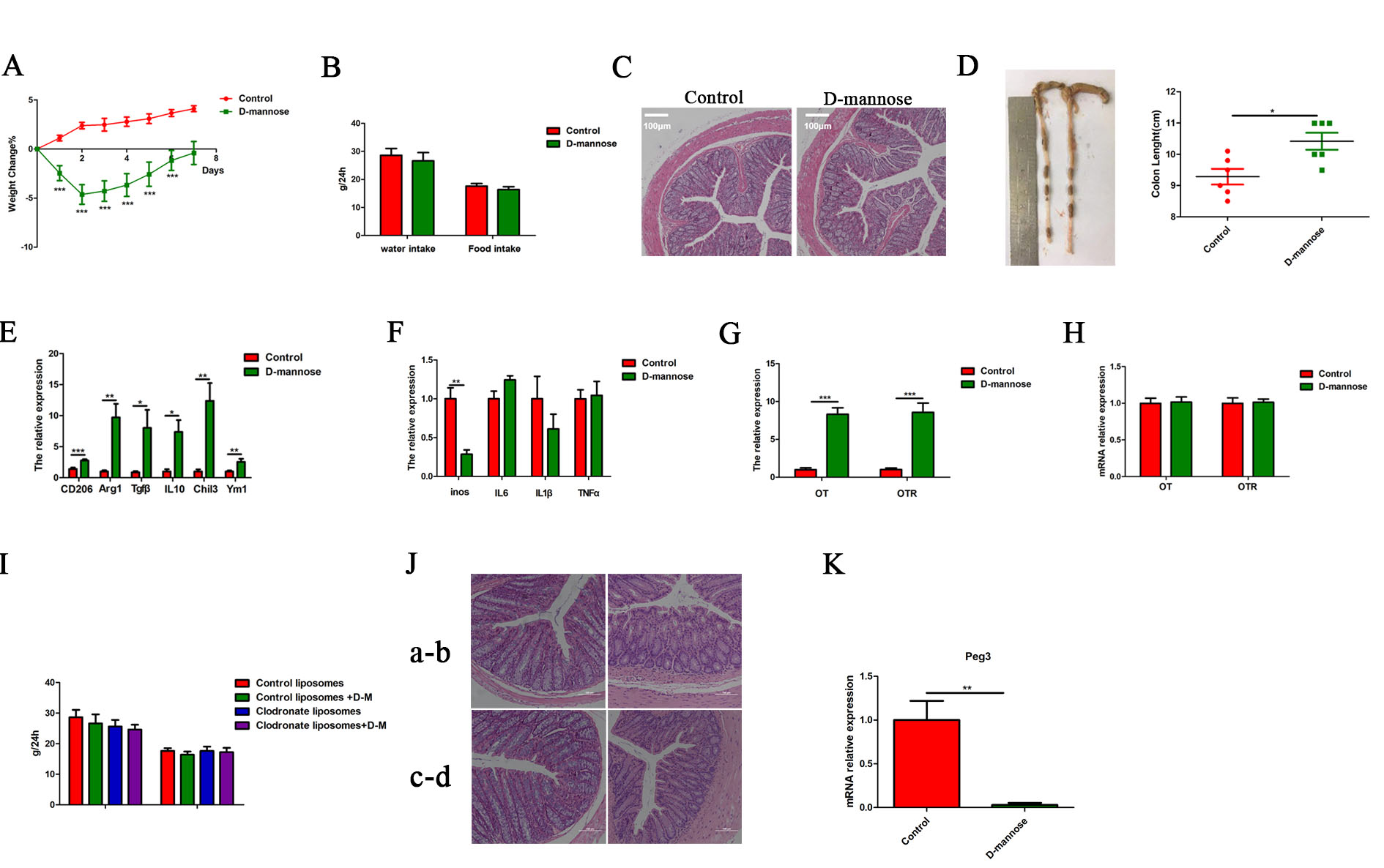

Supplement: Supplementary file 4 — Additional file 4. Fig. S4. D-Mannose promoted polarization of M2 macrophages and inhibited the polarization of M1 macrophages. (A, B) Changes in weight and eating habits in D-mannose model. (C) Representative H&E staining colonic section in each group (Scale bar: 100μm). (D) The lengths of the colon from different groups were statistically compared. (E) The levels of CD206, Arg1, TGF-β, IL10, Chil3, YM1 mRNA of LMMP induced by D-mannose for 7 days were measured by qRT-PCR. (F) The levels of iNOS, IL-1β, IL-6, TNF-α mRNA of LMMP induced by D-mannose for 7 days were measured by qRT-PCR. (G) The levels of OT and OTR mRNA of LMMP induced by D-mannose for 7 days were measured by qRT-PCR. (H) The levels of OT and OTR mRNA by D-mannose for 24 h in enteric neurons were measured by qRT-PCR. (I) Eating habits of mice had no change during the 7 days D-mannose model. (J) The level of Peg3 mRNA of LMMP in D-mannose model was measured by qRT-PCR. The expression of Peg3 of LMMP in the D-mannose group was significantly down-regulated compared with the control group (K) The level of Peg3 mRNA of LMMP in D-mannose model was measured by qRT-PCR. The expression of Peg3 of LMMP in the D-mannose group was significantly down-regulated compared with the control group. The values represent the mean ± SEM of 6 samples and were compared by t-test for multiple comparisons. *p < 0.05, ** p<0.01, *** p<0.001 vs. control group. [file 12974_2021_2313_MOESM4_ESM.jpg]

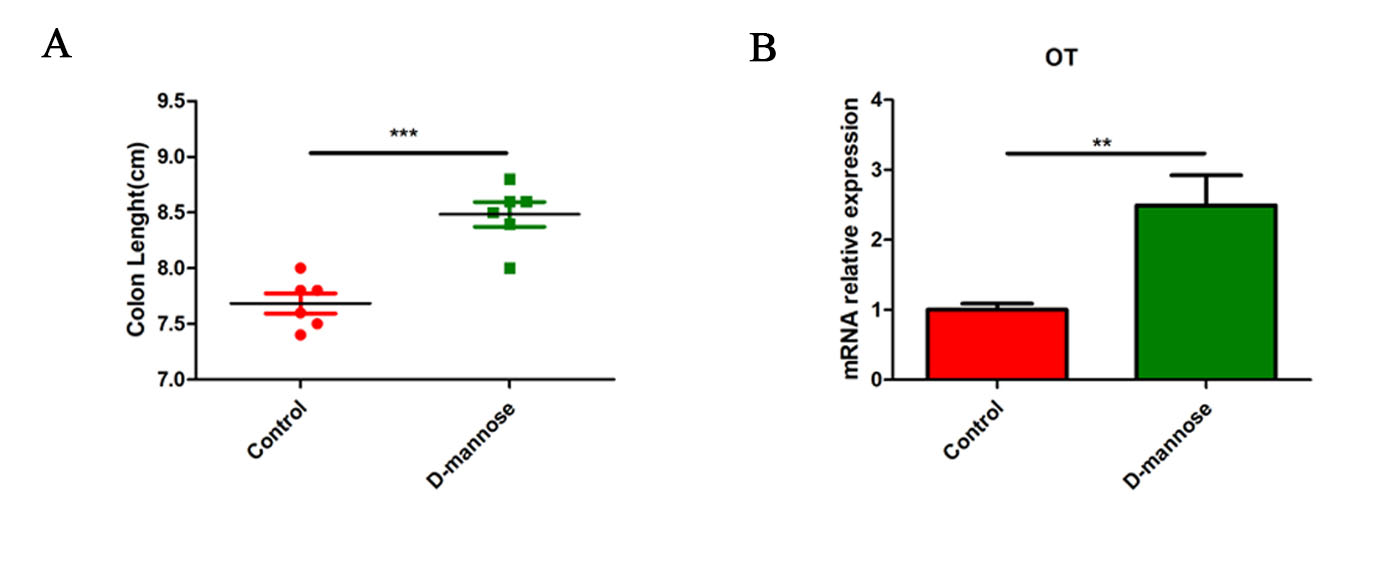

Supplement: Supplementary file 5 — Additional file 5. Fig. S5. The expression of OT was significantly increased in OVX-female mice after drinking D-mannose for 7 days. (A) The change of colon length in female mice. (B) The level of OT mRNA was detected by QPCR in OVX-female mice after drinking D-mannose for 7 days. The values represent the mean ± SEM of 6 samples and were compared by t-test for multiple comparisons. **p<0.01, *** p<0.001. [file 12974_2021_2313_MOESM5_ESM.jpg]
